# Sensory Abnormalities and Pain in the Donor Sites of Radial Forearm and Fibula Free Flaps—A Systematic Review

**DOI:** 10.3390/medicina62081581

**Published:** 2026-08-18

**Authors:** Dinko Martinovic, Slaven Lasic, Ema Puizina, Lovre Martinovic, Jasna Puizina, Josko Bozic, Emil Dediol

**Affiliations:** 1 Department of Maxillofacial Surgery, University Hospital of Split, 21000 Split, Croatia; 2 Department of Maxillofacial Surgery, University of Split School of Medicine, 21000 Split, Croatia; 3 Department of Neurology, University Hospital of Dubrava, 10000 Zagreb, Croatia; 4 Medical Studies, University of Split School of Medicine, 21000 Split, Croatia; 5 Department of Biology, Faculty of Science, University of Split, 21000 Split, Croatia; 6 Department of Pathophysiology, University of Split School of Medicine, 21000 Split, Croatia; 7 Department of Maxillofacial Surgery, University Hospital of Dubrava, 10000 Zagreb, Croatia; emildediol@yahoo.com; 8 School of Medicine, University of Zagreb, 10000 Zagreb, Croatia

**Keywords:** radial forearm free flap, fibula free flap, donor site morbidity, pain, sensory abnormalities

## Abstract

*Background and Objectives*: Free flap reconstruction is the gold standard for complex head and neck defects following oncological ablation. While surgical techniques have improved flap success rates, donor site sensory morbidity remains relatively understudied. The radial forearm free flap (RFFF) and fibula free flap (FFF) are the two most commonly used free flaps for head and neck reconstruction and sensory abnormalities at their donor sites represent a significant but underrecognized source of postoperative morbidity. *Materials and Methods*: The aim of this study is to systematically review and synthesize the existing evidence on the prevalence, characteristics and assessment methods of sensory abnormalities and pain at the donor sites of RFFF and FFF harvests. A systematic review was conducted according to PRISMA 2020 guidance. PubMed/MEDLINE, Scopus, Web of Science, the Cochrane Library, and Google Scholar were searched from inception to 1 October 2025, with additional gray-literature and reference-list screening. Primary studies reporting sensory abnormalities, donor-site pain, neuropathic pain, or cold intolerance after RFFF and/or FFF harvest were included; prior reviews were used for citation tracking and contextual comparison only. Risk of bias was assessed with study-design-specific Joanna Briggs Institute (JBI) checklists, and the level of evidence was classified using the Oxford Centre for Evidence-Based Medicine (OCEBM) framework. Descriptive prevalence summaries and exploratory random-effects pooled estimates were calculated when numerators and denominators could be extracted. *Results*: From 1247 initially identified records, 73 studies met the inclusion criteria (36 RFFF-specific, 35 FFF-specific, and 2 addressing both flap types). Most studies were observational, and sensory outcomes were frequently secondary endpoints. For RFFF, reported sensory abnormalities ranged from 0% to 100% across extractable studies (median 31.3%; IQR 13.1–55.0%), reflecting differences in definitions, follow-up, and testing; the exploratory pooled prevalence was 34.4% (95% CI 23.6–47.1%; I2 = 88.4%). General donor-site pain ranged from 0% to 36.8% (median 12.0%; pooled 16.0%, 95% CI 9.9–24.8%), whereas Douleur Neuropathique en 4 Questions (DN4)-defined neuropathic pain was 18% in the largest dedicated RFFF study and 18% in a smaller pain-focused study. Cold intolerance ranged from 0% to 40.0% (median 16.2%). For FFF, sensory abnormalities ranged from 0% to 76.3% (median 21.4%; IQR 8.2–46.8%; pooled 24.0%, 95% CI 16.5–33.6%; I2 = 88.6%). General pain ranged from 0% to 72.7% (median 17.5%; pooled 18.8%, 95% CI 13.9–24.9%), and DN4-defined neuropathic pain was 21% in the only dedicated FFF study. Objective testing (quantitative sensory testing [QST], Semmes-Weinstein monofilaments, two-point discrimination, or standardized neurological examination) was used inconsistently, and no included study systematically used nerve conduction studies or electromyography (EMG). *Conclusions*: Sensory abnormalities and pain after RFFF and FFF harvest appear common, but available estimates remain uncertain because of heterogeneous definitions, follow-up intervals, assessment instruments, and predominantly Level 2b-4 evidence. The literature shows a persistent gap between patient-reported symptoms and objective sensory testing. Future prospective studies would benefit from standardized neuropathic pain screening, structured neurological examination, and QST; however, current data do not support strong causal inferences or a validated treatment algorithm.

## 1. Introduction

In the last few decades, free flaps have risen to become the gold standard for the reconstruction of defects in the head and neck region, especially after ablative oncological resections [1]. Together with the rise of their popularity, surgical techniques for their harvest have been refined and improved to the point that today a broad range of possible free flaps exists, with each flap having numerous possible modifications depending on the reconstruction requirements [2,3]. Depending on the type of tissue needed, it is possible to add skin, muscles, bone, or even nerves to the main constitution of the flap, which improves both the functional and aesthetic result of the reconstruction. While this is important for the whole human body, it is especially crucial for the head and neck region, which consists of multiple small functional and aesthetic units important for the patient’s quality of life and psychological wellbeing [4].

With the improvement of flap results due to the perfection of harvest techniques and the minimization of flap failures, the focus has lately shifted to the morbidity of the donor sites [5,6,7]. While functionality has improved with free flaps, donor site morbidity remains relatively unexplored. Among all possible free flaps, two are most frequently used in head and neck reconstruction: the radial forearm free flap (RFFF) and the fibula free flap (FFF).

Several systematic reviews have addressed donor-site morbidity following RFFF or FFF harvest [8,9,10,11,12,13]. Accordingly, the novelty of the present review is not that donor-site morbidity has not been reviewed previously, but that sensory abnormalities, donor-site pain, neuropathic pain screening, cold intolerance, and the discordance between subjective and objective sensory assessment methods are evaluated together for both RFFF and FFF. This review therefore updates and integrates the literature by mapping outcome definitions, assessment tools, prevalence estimates, risk of bias, and methodological gaps across the two most frequently used donor sites in head and neck reconstruction.

## 2. Materials and Methods

### 2.1. Study Design

This systematic review was conducted in accordance with the Preferred Reporting Items for Systematic Reviews and Meta-Analyses (PRISMA) 2020 guidance (Supplementary Table S1). The review protocol was developed a priori and registered at the Open Science Framework (https://doi.org/10.17605/OSF.IO/4VXZW). A completed PRISMA 2020 checklist is provided in Supplementary File S1.

### 2.2. Search Strategy

A comprehensive bibliographic search of electronic databases was conducted from inception to 1 October 2025. The following databases were searched: PubMed/MEDLINE, Scopus, Web of Science, the Cochrane Library, and Google Scholar. Full database-specific search strings, search dates, filters, and Google Scholar/gray-literature retrieval details are provided in Supplementary File S1. Google Scholar was searched with prespecified simplified strings, and the first 200 records per string sorted by relevance were screened. Gray-literature screening included indexed theses/dissertations, clinical trial registries, and citation tracking from prior systematic reviews.

### 2.3. Search Terms

The core search concept combined flap-related terms, donor-site terms, and sensory/pain terms. The simplified Boolean structure was: (“radial forearm free flap” OR “RFFF” OR “fibula free flap” OR “FFF” OR “free fibula flap” OR “free tissue transfer”) AND (“donor site” OR “donor-site” OR “donor site morbidity”) AND (“sensory abnormality” OR “sensory deficit” OR “sensory disturbance” OR “hypoesthesia” OR “numbness” OR “paresthesia” OR “dysesthesia” OR “neuropathic pain” OR “pain” OR “neuropathy” OR “cold intolerance”). Database-specific translations of this strategy are reported in Supplementary File S1.

### 2.4. Inclusion and Exclusion Criteria

Studies were included if they reported original data on sensory abnormalities, sensory deficits, neuropathic pain, donor-site pain, or cold intolerance following RFFF and/or FFF harvest; included at least 10 patients; and were published in peer-reviewed journals or indexed gray literature. Eligible primary designs included randomized controlled trials, prospective or retrospective cohorts, cross-sectional studies, comparative studies, and case series meeting the sample-size threshold. Prior systematic reviews and meta-analyses were not included as units of analysis in the evidence table to avoid double counting; they were used for citation tracking, comparison with previous literature, and contextual framing.

Studies were excluded if they were published in a language other than English; were case reports or case series with fewer than 10 patients; did not report donor-site sensory or pain outcomes separately for RFFF or FFF; focused only on recipient-site outcomes; or represented duplicate publication of the same dataset without additional relevant donor-site information.

### 2.5. Study Selection Process

**Figure 1:**
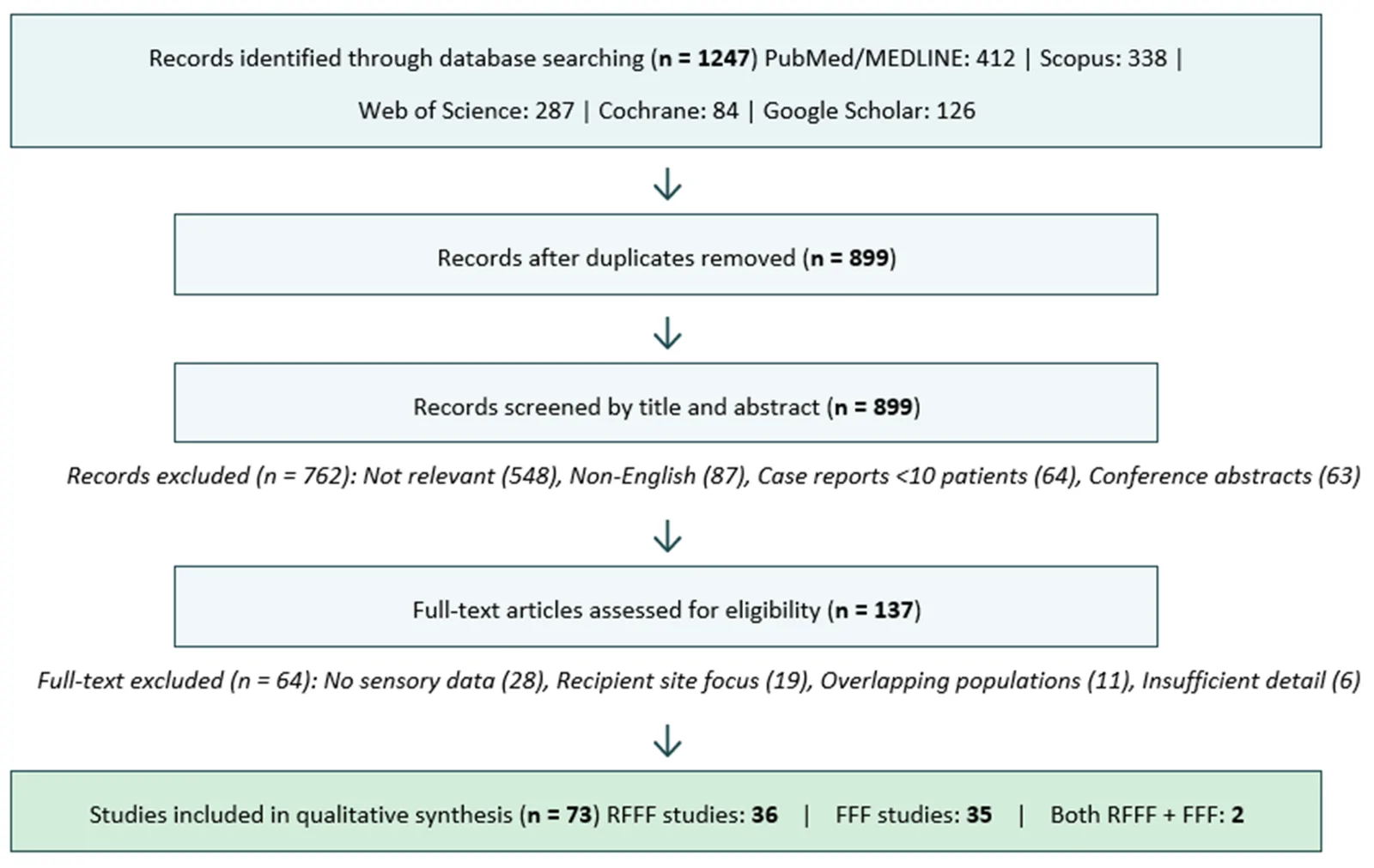
PRISMA flowchart.

Two reviewers independently screened titles and abstracts of all identified records against the inclusion and exclusion criteria. Full-text articles of potentially relevant studies were retrieved and independently assessed for eligibility. Disagreements were resolved through discussion and consensus with a third reviewer when necessary. The study selection process is presented in the PRISMA flow diagram (Figure 1).

### 2.6. Data Extraction

Data were extracted independently by two reviewers using a standardized data extraction form. Extracted variables included study authors and year, design, sample size, flap type (RFFF, FFF, or both), follow-up period, donor-site closure technique when reported, subjective and objective assessment methods, key sensory outcomes (pain, hypoesthesia, paresthesia, dysesthesia, cold intolerance, neuropathic pain, and other sensory abnormalities), whether sensory morbidity was a primary or secondary endpoint, identified risk factors, ethical approval/informed consent reporting, and possible cohort overlap.

### 2.7. Outcome Definitions and Handling of Overlapping Cohorts

Before synthesis, outcomes were operationalized as follows: pain referred to any donor-site pain without necessarily neuropathic features; neuropathic pain required a validated neuropathic pain instrument or explicit neuropathic descriptors; hypoesthesia/numbness indicated reduced sensation; paresthesia indicated non-painful abnormal sensations; dysesthesia indicated unpleasant abnormal sensations; cold intolerance referred to cold-provoked discomfort, pain, or altered temperature tolerance; and sensory deficit/abnormality was used as an umbrella term. When studies used different terminology, the authors’ original outcome was extracted and then mapped to these categories for synthesis. This mapping reduces but does not eliminate misclassification because many studies did not provide protocol-defined sensory outcomes.

Potentially overlapping cohorts were handled conservatively. Overlapping reports were retained in the qualitative table when they added distinct outcomes or follow-up information, but only the most complete, largest, or longest follow-up dataset was used in quantitative summaries to reduce double counting. Known or suspected overlap was flagged in Table 1 or Supplementary Table S2.

### 2.8. Quality, Level-of-Evidence, and Risk-of-Bias Assessment

The level of evidence of each included study was classified according to the Oxford Centre for Evidence-Based Medicine (OCEBM) 2011 Levels of Evidence framework. In addition, formal risk-of-bias assessment was performed independently by two reviewers using study-design-specific Joanna Briggs Institute (JBI) critical appraisal checklists (randomized trial, cohort, analytical cross-sectional, and case-series tools as applicable). Disagreements were resolved by consensus. Overall risk of bias was graded as low, moderate, or high based on selection, outcome measurement, follow-up completeness, confounding, and reporting transparency. OCEBM levels were used as an evidence hierarchy and were not considered a substitute for risk-of-bias assessment.

Level 1a: Systematic reviews (with homogeneity) of randomized controlled trials

Level 1b: Individual randomized controlled trials (with narrow confidence intervals)

Level 2b: Individual cohort studies or low-quality randomized controlled trials

Level 3: Individual case-control studies, cross-sectional studies, or retrospective comparative studies

Level 4: Case series (and poor-quality cohort and case-control studies)

Level 5: Expert opinion without explicit critical appraisal, or based on physiology, bench research, or first principles

Primary studies were also checked for reporting of ethical approval and informed consent. These variables were extracted when available but were not used as exclusion criteria because reporting standards differed substantially across decades.

### 2.9. Data Synthesis and Quantitative Summary

Qualitative synthesis was considered primary because the included studies differed substantially in design, follow-up interval, donor-site closure technique, outcome definitions, and sensory assessment methods. For outcomes with extractable numerators and denominators, reported ranges, medians, and interquartile ranges (IQRs) were calculated. Exploratory random-effects proportional meta-analysis used logit-transformed proportions and DerSimonian-Laird between-study variance; a 0.5 continuity correction was applied to zero-event studies. Heterogeneity was quantified with I2. Pooled estimates are presented as descriptive statistical summaries and interpreted cautiously, particularly when I2 was high. Sensitivity analyses excluded cohorts with fewer than 20 patients and overlapping publications.

## 3. Results

### 3.1. Study Selection

The PRISMA flow diagram (Figure 1) summarizes the study selection process. The initial database search identified 1247 records (PubMed/MEDLINE: 412, Scopus: 338, Web of Science: 287, Cochrane: 84, Google Scholar: 126). After removal of 348 duplicate records, 899 unique records underwent title and abstract screening. Of these, 762 were excluded based on the following criteria: not relevant to sensory outcomes at RFFF/FFF donor sites (n = 548), non-English language (n = 87), case reports or series with fewer than 10 patients (n = 64), conference abstracts without full data (n = 63). Hence, 137 full-text articles were assessed for eligibility, and 64 were subsequently excluded: 28 did not report specific sensory outcome data, 19 focused on recipient site outcomes rather than donor site morbidity, 11 had overlapping patient populations with included studies, and 6 lacked sufficient methodological detail. Ultimately, 73 studies met all inclusion criteria and were included in the qualitative synthesis.

### 3.2. Study Characteristics

The 73 included studies were published between 1986 and 2025. Study designs comprised 4 randomized controlled trials (Level 1b), 24 prospective cohort studies (Level 2b), 39 cross-sectional or retrospective studies (Level 3), and 6 retrospective case series (Level 4). Sample sizes ranged from 10 to 361 patients. RFFF was exclusively assessed in 36 studies, FFF in 35 studies, and both flap types in 2 studies. Detailed study characteristics are presented in Table 1; risk-of-bias judgments and donor-site closure techniques are summarized in Supplementary Tables S2 and S3, respectively.

### 3.3. Sensory Abnormalities After RFFF Harvest

The synthesized evidence from the studies which addressed sensory abnormalities at the RFFF donor site revealed substantial sensory morbidity concentrated predominantly in the superficial radial nerve territory.

#### 3.3.1. Prevalence of Sensory Deficits

RFFF hypoesthesia rates varied widely across studies using explicit hypoesthesia outcomes, ranging from 11% to 89%. When broader extractable sensory abnormality outcomes were included (e.g., numbness, paresthesia, abnormal monofilament thresholds, or objective disturbance area), reported estimates ranged from 0% to 100% (median 31.3%, IQR 13.1–55.0%). The exploratory random-effects pooled prevalence for extractable RFFF sensory abnormalities was 34.4% (95% CI 23.6–47.1%; I2 = 88.4%), indicating substantial heterogeneity. The highest explicit hypoesthesia rates were reported by Lee et al. at 89% and Loreti et al. at 88%, both in small cohorts (n = 19 and n = 17, respectively) [16,17]. Toschka et al. documented hypoesthesia in 77.1% using objective tools including two-point discrimination and stereognosis testing [28]. More moderate rates were reported by de Bree et al. at 46%, Lane et al. at 42%, and Riecke et al. at 40%, while larger or longer-term studies reported lower rates: Thiem et al. found hypoesthesia in 27% of the RFFF arm, Deneuve et al. in 14.3% of 191 patients at mean 34 months, and Riecke et al. in 20% at long-term follow-up [15,18,34,35,41,42]. Novak et al. reported numbness in 42% but formal hypoesthesia in 11% [20].

Wang et al. (2013) reported a paresthesia rate of 88.6%, although this was a secondary outcome from a study primarily designed to assess ALT flap superiority and should be interpreted cautiously [38]. Cold intolerance ranged from 0% to 40.0% across extractable RFFF studies (median 16.2%, IQR 8.0–26.7%; exploratory pooled prevalence 20.1%, 95% CI 13.0–29.9%). The highest RFFF-specific rate was reported by Toschka et al. at 31.4%, followed by Timmons et al. at 27%, Novak et al. at 26%, Lane et al. and de Bree et al. at 16%, and Faisal et al. at 8% [15,18,20,25,28,33]. The higher 40% estimate came from a small mixed-flap study and was retained for qualitative completeness [60].

Sensory deficits were consistently concentrated in the superficial radial nerve (SRN) territory across studies (Loreti et al., Lane et al., Grinsell et al., Kerawala & Martin, Kitano et al., Rashid et al.) [15,17,19,24,47,60].

#### 3.3.2. Pain and Neuropathic Pain

General donor-site pain was reported in 0% to 36.8% of extractable RFFF primary cohorts (median 12.0%, IQR 0–20.0%; exploratory pooled estimate 16.0%, 95% CI 9.9–24.8%; I2 = 56.6%). Novak et al. reported the highest pain rate at 36.8% (7/19), while Lee et al. reported pain in 26% and Pirlich et al. reported donor-site pain in 18% with neuropathic-type features [16,20,23]. Deneuve et al. documented chronic pain in 13.8% of 191 patients at mean 34 months, and Hong reported scar pain in 10% of 67 patients [41,43]. Several prospective RFFF cohorts reported no chronic pain at later follow-up, underscoring the importance of follow-up interval and pain definition [34,35,36].

Neuropathic pain assessed with the Douleur Neuropathique en 4 Questions (DN4) questionnaire was identified by Bruin et al. in 18% of 114 RFFF patients and was significantly associated with wound complications (tendon exposure, 32%), reduced quality of life (*p* < 0.001), and impaired hand function (*p* < 0.001) [14]. Pirlich et al. independently identified neuropathic-type pain features in 18% of 39 patients [23]. Because only two RFFF studies used neuropathic-pain-specific screening, this estimate should be interpreted as a focused finding rather than a stable population prevalence.

#### 3.3.3. Temporal Recovery Pattern

Richardson et al., the only primary study in this review providing systematic time-point data (n = 86), demonstrated a declining trajectory: 75% of patients had reduced sensation at 3 months, decreasing to 50% at 6 months and 32% at 12 months [44]. However, Wirthmann et al. found that sensory deficits persisted in 54% of patients at a mean follow-up of 24 months, a rate seemingly higher than the 12-month figure from Richardson et al. [45]. This apparent paradox likely reflects differences in patient populations, harvest techniques, and—critically—the assessment method used, as Wirthmann et al. employed patient questionnaires without objective validation [45]. Byun et al. documented a measurable sensory disturbance area in all 10 patients at 6 months (mean 18.70 ± 4.39 cm^2^), while Riecke et al. (2015) found persistent thenar hypoesthesia in 20% at mean 2.2 years [34,36].

These data collectively suggest that while the majority of patients experience partial recovery within the first year, a clinically important minority—possibly one-third to over half depending on assessment method—retains measurable sensory deficits beyond 12–24 months.

#### 3.3.4. Risk Factors

Hong identified hypertension, diabetes, and advanced age as significant predictors of donor site sensory complications in 67 RFFF patients [43]. Diabetes carried elevated risk for paresthesia, while acellular matrix dressing use was associated with reduced paresthesia prevalence [43]. Kitano et al. (2023) demonstrated that preservation of the superficial branch of the radial nerve significantly improved both subjective and objective sensory outcomes (*p* = 0.019 and *p* = 0.0035, respectively) [24]. Grinsell et al. (2005) demonstrated that the approach to venous harvest significantly influenced SRN morbidity, as raising the cephalic vein doubled the incidence of SRN sensory deficit compared to using the venae comitantes (18% vs. 9%), highlighting surgical technique as an important modifiable risk factor [19].

### 3.4. Sensory Abnormalities After FFF Harvest

Studies examining sensory outcomes at the FFF donor site also showed substantial variability in reported incidence rates, largely reflecting differences in terminology, follow-up interval, and whether objective neurological testing was performed.

#### 3.4.1. Prevalence of Sensory Deficits

Sensory deficit rates after FFF harvest showed the widest variation of all outcomes in this review, ranging from 0% to 76.3% across extractable studies (median 21.4%, IQR 8.2–46.8%). The exploratory random-effects pooled prevalence was 24.0% (95% CI 16.5–33.6%; I2 = 88.6%), again demonstrating substantial heterogeneity rather than a precise single estimate.

The highest objective sensory deficit rate was documented by Zimmermann et al., who found objective sensory deficits in 76.3% of 38 patients using standardized bilateral comparison with cotton swab and pressure probe testing, including touch hypoesthesia in 60.5%, pressure hypoesthesia in 50%, and anesthesia in 36.8% [70]. Xu et al. reported lateral calf numbness in 73.3% of 30 patients at 6 months [54]. Celotti et al. documented sensory deficits in 50% and Bodde et al. in 50% of patients [58,73]. Sieg et al. found dysesthesia in 48% of 62 donor sites using standardized neurological examination with involvement of the sural nerve territory in 26%, superficial peroneal nerve in 31%, and deep peroneal nerve in 32% [80]. Rendenbach et al. reported sensory deficits (paresthesia, anesthesia, hypoesthesia) in 48.1% of 27 patients [84]. De Lange et al. found hypoesthesia in 49% and paresthesia in 28% among 82 patients using the DN4 and validated questionnaires [50]. At the lower end, Babovic et al. reported foot numbness in 10% of 100 patients, Ali et al. reported early sensory deficits in 5.3% of 361 patients, Chou et al. found sensory impairment in 9.1% of 11 patients, and Attia et al. reported paresthesia in 7.3% of 68 patients [53,57,68,79]. Three studies reported 0% objective sensory deficit (Hidalgo et al., Shpitzer et al., Ling et al.), but all three used non-standardized assessment tools or qualitative examinations that may have underestimated subtle sensory change [62,66,83].

Cold intolerance in FFF patients ranged from 0% to 61.1% across extractable studies (median 6.7%, IQR 2.2–33.5%; exploratory pooled prevalence 15.0%, 95% CI 5.4–35.2%; I2 = 82.0%). Celotti et al. reported the highest rate at 61.1% (n = 18), Rashid et al. reported 40% in a small mixed-flap cohort, De Lange et al. reported 17%, and Xu et al. reported 6.7% [50,54,58,60]. Anthony et al. and Shpitzer et al. reported 0%, emphasizing the influence of assessment method and follow-up duration [63,66].

#### 3.4.2. Pain and Neuropathic Pain

General pain rates after FFF harvest varied considerably, ranging from 0% to 72.7% in extractable studies (median 17.5%, IQR 7.1–27.6%; exploratory pooled prevalence 18.8%, 95% CI 13.9–24.9%; I2 = 77.7%). Feuvrier et al. reported the highest pain rate at 72.7% (n = 11, mean 28 months), followed by Bodde et al. at 60% (n = 10) and Garrett et al. at 42.9% (n = 14), although these were small cohorts [73,74,85]. In larger samples, Ali et al. reported chronic pain in 8.0% of 361 patients, Pototschnig et al. moderate pain in 8.7% of 104 patients, and Attia et al. persistent pain in 4.4% of 68 patients [51,53,57]. Intermediate estimates included Shindo et al. (29.6%), Zimmermann et al. (23.7%; NRS mean 4.4 ± 1.5), Bartaire et al. (23%), and Di Giuli et al. (21%) [52,56,69,70].

Neuropathic pain assessed by DN4 was documented in 21% of 82 FFF patients by de Lange et al., the only dedicated neuropathic-pain study in FFF patients. Associated symptoms included hypoesthesia (49%), paresthesia (28%), itching (23%), stinging (23%), burning (18%), cold intolerance (17%), and electrical shock sensations (12%), with a median VAS pain score of 5.0 [50]. Because this outcome currently rests on one dedicated study, the 21% figure should be considered an important signal requiring replication rather than a definitive pooled prevalence.

#### 3.4.3. Dissociation Between Subjective and Objective Findings

A particularly noteworthy finding was the dissociation between subjective complaints and objective sensory measurements. Bartaire et al. evaluated 23 FFF patients and found that while 23% reported pain requiring analgesics, objective testing revealed preserved tactile sensitivity, normal two-point discrimination, and intact pain sensation [52]. This finding highlights the limitations of relying solely on either subjective or objective assessment methods and underscores the need for comprehensive multimodal evaluation.

### 3.5. Assessment Methods Used

The Douleur Neuropathique en 4 Questions (DN4) questionnaire, designed specifically to identify neuropathic pain, was employed only by Bruin et al. for RFFF and de Lange et al. for FFF [14,50].

Far fewer studies employed objective sensory testing. Semmes-Weinstein monofilaments were used in 6 studies, while two-point discrimination was performed in 10 studies. Comprehensive quantitative sensory testing (QST), including thermal stimulation, weighted pinprick stimulators, and digital pressure algometry, was performed in only 1 study (Wang et al. 2018) [39]. Gait analysis was used in 2 FFF studies (Di Giuli et al., Xu et al.) [54,56]. Nerve conduction studies and EMG were not systematically employed in any included study.

### 3.6. Level of Evidence Analysis

The level of evidence across the 73 included studies was predominantly moderate to low. Four studies (5.5%) were classified as Level 1b randomized controlled trials, 24 studies (32.9%) as Level 2b prospective cohorts, 39 studies (53.4%) as Level 3 cross-sectional or retrospective studies, and 6 studies (8.2%) as Level 4 retrospective case series. No Level 1a reviews were included as units of analysis. None of the Level 1b randomized trials specifically investigated sensory outcomes as a primary endpoint. Thiem et al. compared RFFF with ulnar forearm flaps, while Clark et al. compared negative pressure with static pressure wound dressings; in both trials, sensory outcomes were secondary endpoints [42,48].

### 3.7. Risk-of-Bias Assessment

Formal risk-of-bias assessment showed that the evidence base was methodologically limited despite the large number of included studies. Overall, 9 studies were judged to be at low risk of bias, 42 at moderate risk, and 22 at high risk (Supplementary Table S2). The most frequent limitations were retrospective or cross-sectional design, non-consecutive sampling, variable or incompletely reported follow-up, sensory outcomes recorded as secondary endpoints, non-validated patient-reported instruments, absence of preoperative or contralateral comparison, and limited control of confounding. These judgments did not lead to study exclusion, but they were used to temper the certainty of the prevalence conclusions.

### 3.8. Quantitative Prevalence Summary and Sensitivity Analysis

**Figure 2:**
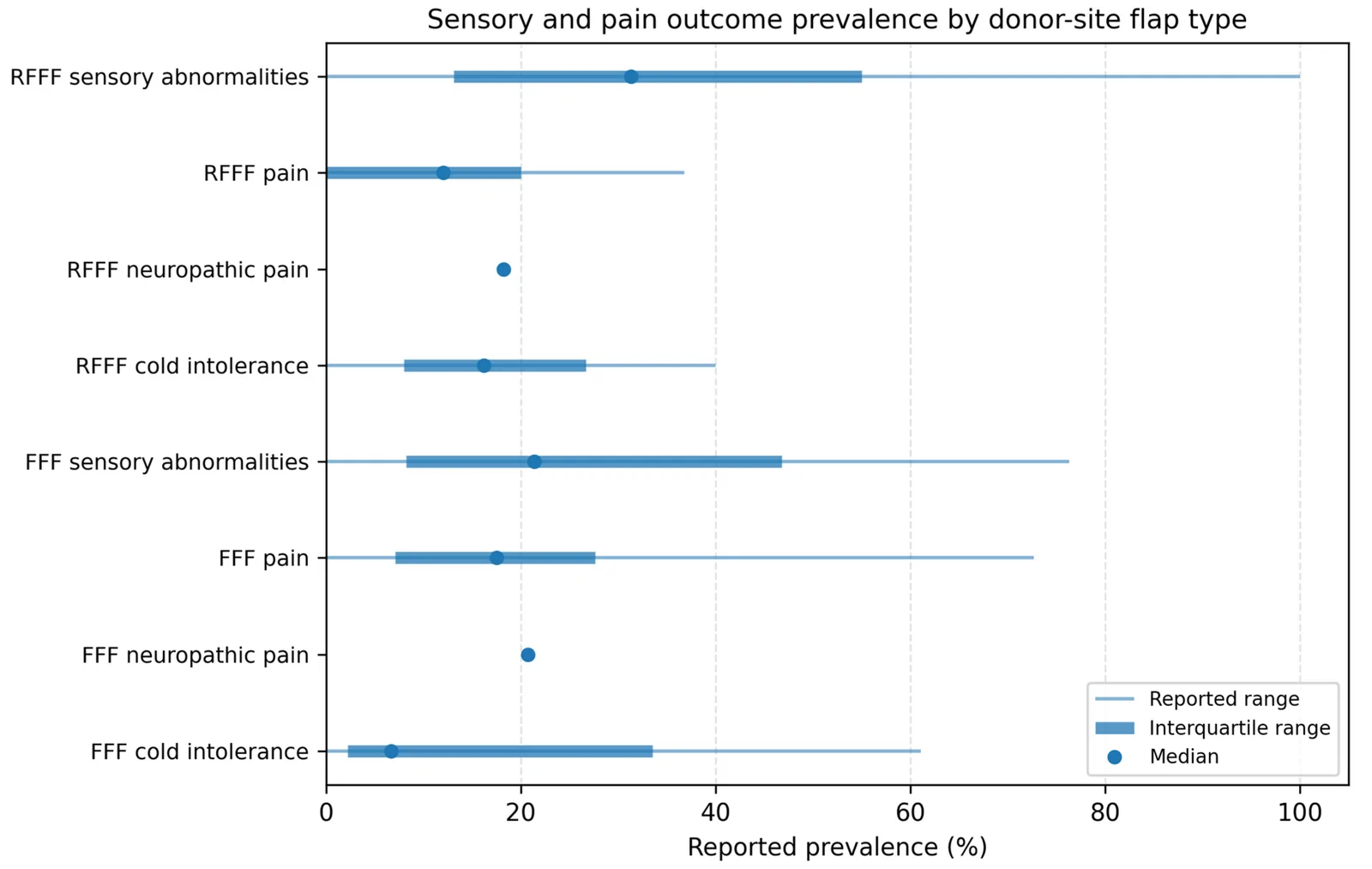
Reported prevalence ranges, interquartile ranges, and medians for extractable sensory and pain outcomes by flap type.

Exploratory quantitative synthesis was feasible only for outcomes with extractable numerators and denominators. The resulting pooled estimates had substantial heterogeneity for most sensory outcomes, so they are best interpreted alongside medians, IQRs, and ranges rather than as definitive population prevalences. Sensitivity analyses excluding cohorts with fewer than 20 patients generally shifted estimates downward for sensory abnormalities and pain, especially in RFFF sensory outcomes and FFF pain, but did not resolve heterogeneity (Supplementary Table S5). The quantitative summary is shown in Table 2 and Figure 2.

## 4. Discussion

This systematic review updates and integrates the current evidence on sensory abnormalities and pain at RFFF and FFF donor sites across 73 studies. The findings suggest that sensory morbidity is a common but variably measured complication of both donor sites, although the certainty of prevalence estimates is limited by heterogeneous outcome definitions, follow-up intervals, assessment methods, and the predominance of observational evidence.

Compared with prior systematic reviews, the present study should be interpreted as a focused update and methodological synthesis rather than as the first review of donor-site morbidity (Supplementary Table S4). Bruin et al. [8] focused on RFFF neuropathic pain and altered sensation; Yalamanchili et al. [9] and Loeffelbein et al. [11] primarily addressed broader RFFF donor-site morbidity and closure-related questions; Chen et al. [10] compared ALT and RFFF morbidity in tongue reconstruction; Kearns et al. [12] summarized osteocutaneous flap donor morbidity and patient-reported outcomes; and Ling and Peng [13] synthesized FFF donor-site morbidity broadly. The present review differs by evaluating RFFF and FFF in one framework, extracting sensory and pain outcomes specifically, contrasting subjective and objective assessment modalities, incorporating DN4-based neuropathic pain studies, and adding formal risk-of-bias and quantitative prevalence summaries. Thus, the main contribution is integration, methodological appraisal, and sensory-outcome focus rather than the claim that donor-site morbidity has not been reviewed before.

### 4.1. Anatomical Considerations in RFFF Harvest

The RFFF, originally a fasciocutaneous free flap introduced in the early 1980s by Chinese surgeons, remains one of the most frequently selected free tissue transfers for head and neck reconstruction, particularly when thin, supple soft tissue is required [87]. Sensory perception of the flap territory derives from a triple nerve supply [88]. The lateral antebrachial cutaneous nerve (LABCN) represents the principal sensory source, supplying approximately 60% of the flap’s sensory distribution. Two supplementary nerve sources, the medial antebrachial cutaneous nerve (MABCN) and the superficial radial nerve (SRN), provide additional sensory contributions.

The harvesting procedure involves multiple stages in which sensory structures are at risk [89]. The SRN is vulnerable during exposure of the cephalic vein because the nerve lies in close proximity to the venous dissection plane. The marginal dissection of flap edges may place the LABCN at risk [89]. Mobilization of the brachioradialis muscle to access the radial artery is another potentially vulnerable phase because small perforators and sensory branches may travel in the same operative field. Anatomical variations of the SRN, including variable trajectory patterns and multiple subdivisions, can increase the risk of misidentification and unplanned removal of sensory branches [90]. These anatomical and surgical considerations provide a plausible explanation for the concentration of RFFF sensory deficits in the SRN territory, but the included studies rarely used nerve localization methods strong enough to prove nerve-specific causality.

### 4.2. Anatomical Considerations in FFF Harvest

The FFF, first demonstrated for mandibular reconstruction by Hidalgo in 1989 [62], offers superior length compared to alternative vascularized osseous options, permitting reconstruction of even complete mandibular defects. The skin of the FFF receives cutaneous innervation from multiple overlapping nerve distributions. The lateral sural cutaneous nerve (LSCN) has been regarded as the primary sensory nerve supply, while recent anatomical research has revealed dual innervation through the LSCN and a previously unrecognized distal branch of the superficial peroneal nerve—the recurrent superficial peroneal nerve (RSPN) [91,92].

Multiple nerve territories are at risk during FFF harvest. The superficial peroneal nerve is vulnerable during anterior skin incision and distal-third dissection [53,59]. The lateral sural cutaneous nerve and RSPN branches are relevant during intermuscular septum division. The deep peroneal nerve may be threatened during division of the interosseous membrane [93], and the common peroneal nerve at the fibular head is vulnerable during proximal dissection [94]. These multiple at-risk neural structures help explain the wide range of sensory deficits documented in this review, but the exact nerve injured in an individual patient cannot usually be inferred from the available studies because objective localization was rarely performed.

### 4.3. Pathophysiology of Sensory Symptoms and Pain

Nerve injury during RFFF and FFF harvest may disrupt normal somatosensory function through several mechanisms. Different receptors of peripheral sensory neurons transduce mechanical, thermal, chemical, and noxious signals into action potentials, which are transmitted through pseudounipolar neurons to dorsal spinal columns [95]. Injury of large non-nociceptive fibers (Aβ), such as may occur after RFFF (SRN, LABCN, MABCN) or FFF harvest (RSPN, LSCN), can impair touch, pressure, and proprioception, while small-fiber injury (Aδ and C fibers) can alter pain and temperature sensation (Figure 2) [96].

Neuropathic pain develops after a lesion or disease of the somatosensory nervous system, including peripheral nerve injury. It may be continuous (e.g., burning), provoked (e.g., thermal evoked), or paroxysmal (e.g., electric shock-like) [97]. Flap harvesting and donor-site closure may plausibly cause compressive demyelination, nerve traction, partial transection, neuroma formation, axonal sprouting, sodium-channel upregulation, and ectopic discharges, which can manifest as itching, burning, tingling, electric shock-like pain, hypoesthesia, or dysesthesia [98,99]. The temporal recovery patterns reported after RFFF and FFF harvest are compatible with a spectrum of neuropraxic, axonotmetic, or neurotmetic injuries; however, the included studies did not systematically perform nerve conduction studies or EMG. Therefore, the available evidence does not permit assignment of a specific Seddon or Sunderland grade [100,101]. Any inference regarding injury grade should be considered hypothesis-generating and should not be used to determine surgical exploration thresholds without individualized clinical assessment.

Ongoing burning pain and cold intolerance may reflect small-fiber dysfunction involving C and Aδ fibers, which is better detected by QST or structured bedside sensory testing than by routine large-fiber examination. Conversely, compressive injury to large myelinated Aβ fibers may contribute to mechanically evoked symptoms and paroxysmal shock-like pain and may be better evaluated by nerve conduction studies when clinically indicated [97,102]. Studies that recorded only constant pain or broad discomfort may therefore have missed event-related allodynia, cold-evoked pain, or shock-like pain.

**Figure 3:**
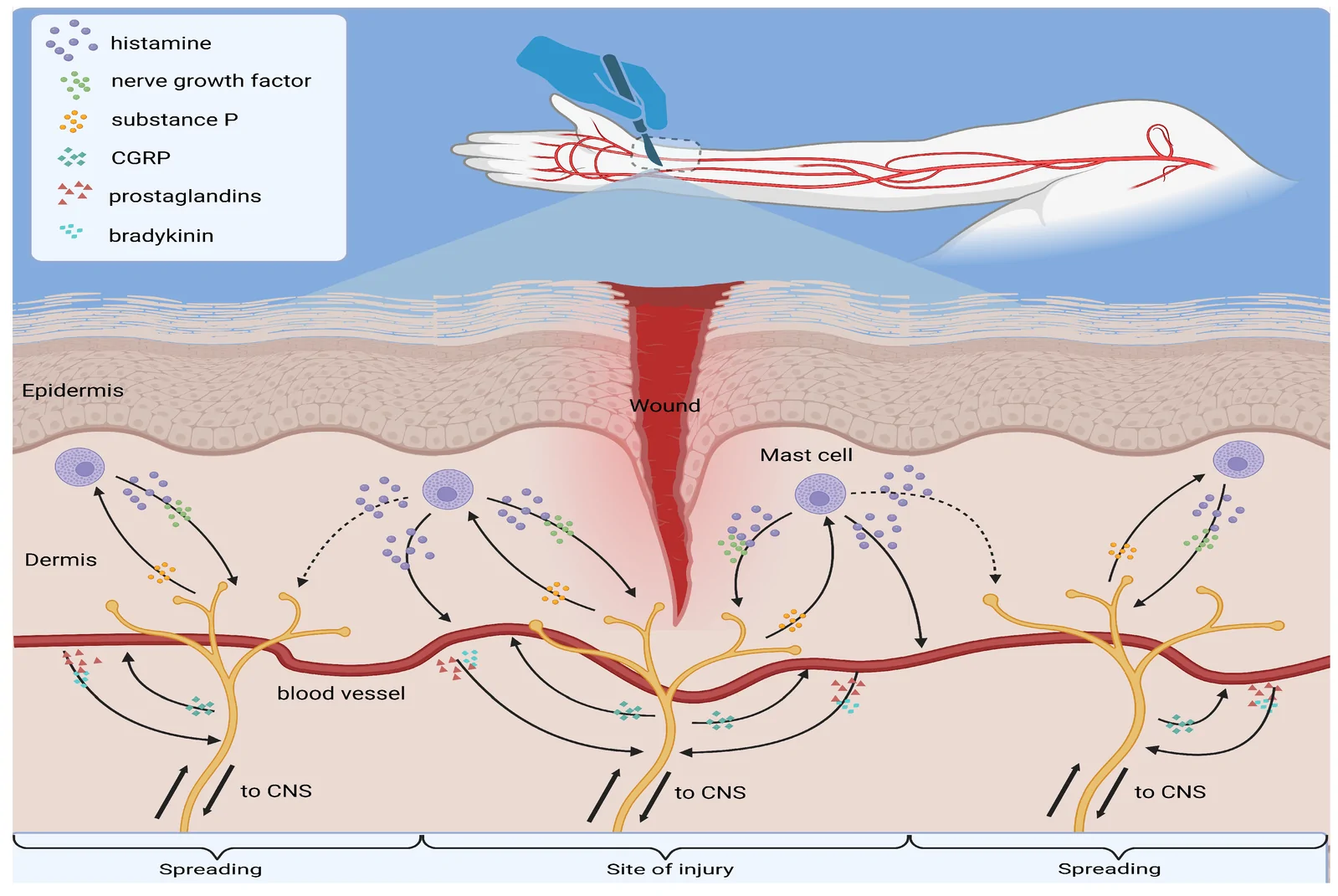
Neurogenic inflammation spreading to painful stimuli.

Additionally, both peripheral and central sensitization mechanisms play important roles. Peripheral sensitization occurs through direct stimulation of Aδ and C nociceptors or through decreased thresholds due to locally released inflammatory mediators (bradykinin, substance P, histamine, ATP, nerve growth factor, prostaglandins) (Figure 3). Central sensitization occurs through C-fiber release of substance P, CGRP, and BDNF, causing “wind up” of dorsal horn neurons and expansion of receptive fields [103].

Previous studies have connected weather sensitivity and cold intolerance at RFFF donor sites to vascular mechanisms, whereas studies by Suominen et al. questioned a purely vascular explanation and proposed disturbed sympathetic tone [104,105]. Partial denervation, peripheral sensitization, TRPM8/TRPA1-related cold transduction, and sympathetically maintained pain may contribute to cold-provoked symptoms [106,107,108]. Accordingly, cold intolerance may overlap mechanistically with neuropathic pain in some patients, but the present donor-site literature does not justify treating it as a direct analogue or diagnostic equivalent of neuropathic pain.

Chronic pain at RFFF and FFF donor sites may arise from peripheral nerve injury, small-fiber dysfunction, peripheral sensitization related to wound complications or closure technique, or nociplastic mechanisms in predisposed patients [109]. The included studies are not sufficient to distinguish these mechanisms reliably. Evidence regarding pain etiology and anatomical localization in most studies remains modest.

#### Surgically Induced Peripheral Neuropathic Pain

**Figure 4:**
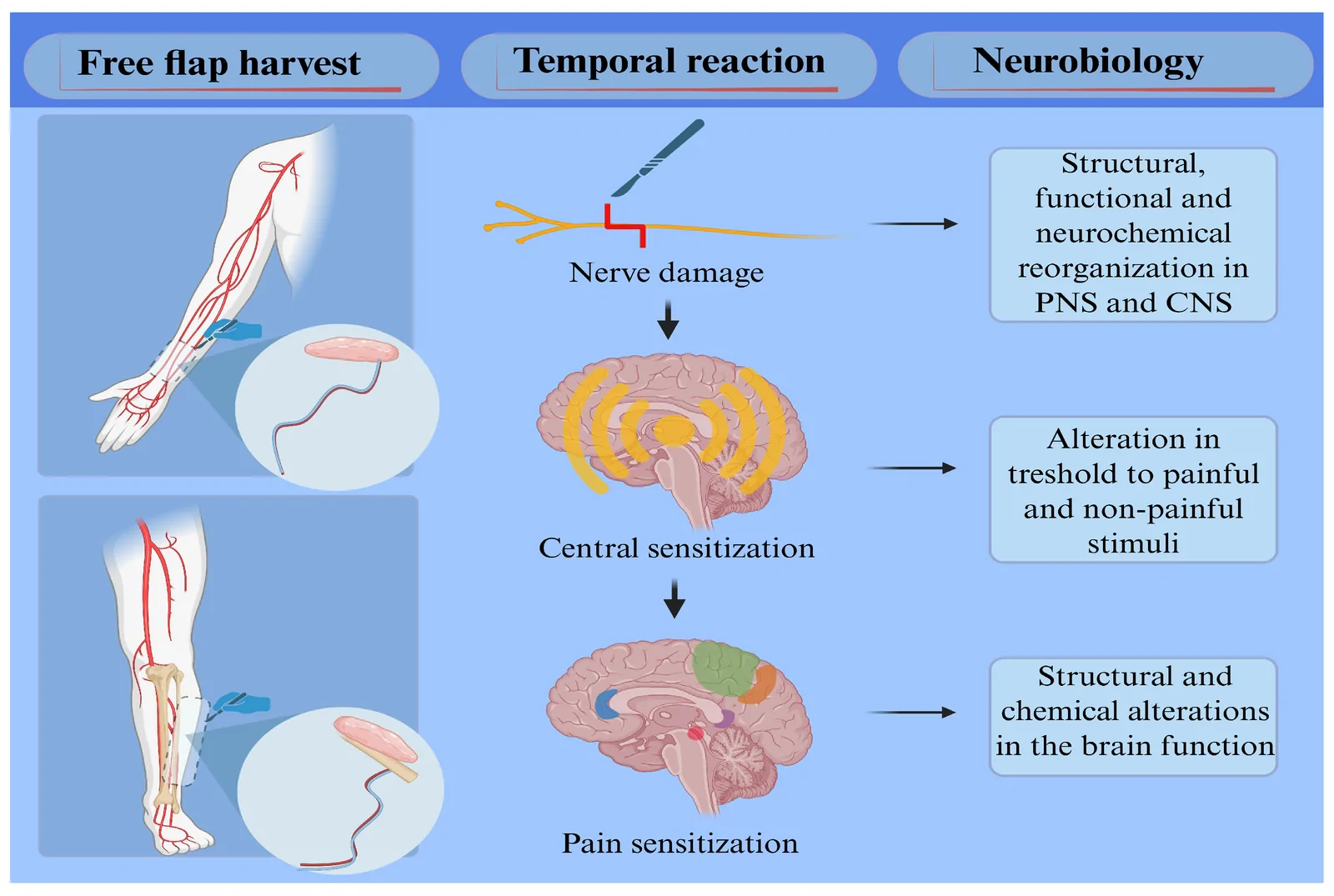
Surgically induced peripheral neuropathic pain.

Among subtypes of peripheral neuropathic pain, surgically induced peripheral neuropathic pain (SPNP) is caused by a deliberate surgical intervention that damages or irritates nerves (Figure 4) [110]. SPNP develops in 10–50% of individuals after common surgeries, with 2–10% reporting severe pain (>4/10 VAS) [111]. Female sex, younger age, severe acute postoperative pain, pre-existing painful states, anxiety, and depression have been associated with elevated risk of chronic postoperative pain [111,112,113].

The DN4-defined neuropathic pain estimates identified in this review (18% after RFFF and 21% after FFF) are broadly consistent with the SPNP literature, but the donor-site evidence remains too sparse to define precise prevalence or risk prediction models. The findings nevertheless support routine attention to donor-site pain quality, sensory change, and functional impact during follow-up. In a rare example of disability-oriented donor-site reporting, Suominen et al. described patients with work disability, retirement, or ongoing disability evaluation related to sensory deficit, pain, or altered grip strength [104].

### 4.4. Gap Between Subjective and Objective Assessment

One of the most significant findings of this systematic review is the marked disparity in assessment methods used across studies. The majority of included studies relied on subjective patient-reported outcomes, unstructured interviews, non-standardized questionnaires, or VAS scores. These measures are clinically important because they capture the patient experience, but they are susceptible to recall bias, variable pain thresholds, and differences in how patients understand terms such as numbness, altered sensation, paresthesia, or pain. In many included studies, sensory morbidity was a secondary endpoint rather than the primary study objective, further limiting precision.

In contrast, objective assessment methods were employed in a minority of studies. Semmes-Weinstein monofilaments were used in 6 studies, two-point discrimination testing in 10 studies, and comprehensive QST in only 1 study. No study systematically employed nerve conduction studies or EMG to localize nerve damage. This is a major limitation because the available evidence cannot definitively demonstrate that specific nerves (SRN, LABCN, MABCN, common peroneal nerve, superficial peroneal nerve, or lateral sural cutaneous nerve) were directly injured during flap harvest and were causally responsible for neuropathic pain. Current literature, including the studies by Bruin et al. and de Lange et al., generally supports no more than probable neuropathic pain because DN4 screening was not combined with objective confirmation of somatosensory system damage [114].

The dissociation between subjective complaints and objective findings, exemplified by Bartaire et al. reporting pain requiring analgesics in 23% of FFF patients despite preserved tactile sensitivity and normal two-point discrimination, underscores the complexity of post-surgical sensory assessment [52]. This discordance may reflect small-fiber dysfunction affecting pain and temperature while sparing large-fiber modalities, central sensitization, or limitations of the objective tests selected. Patient-reported outcome measures (PROMs) and objective sensory testing therefore measure complementary aspects of donor-site morbidity: PROMs capture spontaneous symptoms and quality-of-life impact, while objective testing characterizes provoked responses and sensory loss patterns. Both approaches are needed in future donor-site studies.

### 4.5. Assessment Methods for Clinical Practice

Neuropathic pain is especially important to diagnose because regular analgesic treatment does not adequately control it [115]. When suspecting neuropathic pain, physical examination must confirm sensory or motor deficit. Negative sensory symptoms such as hypoesthesia alongside pain confirm peripheral nervous system dysfunction, while positive symptoms (paresthesia, dysesthesia, allodynia, hyperalgesia) alongside pain suggest both peripheral and potential central sensitization [115].

Questionnaires such as the DN4 were designed to facilitate diagnosis of neuropathic pain and supplement detailed neurological examination [116]. The DN4 is an internationally validated questionnaire that includes 7 questions about neuropathic pain indicators, with a score ≥ 4 indicating neuropathic pain. Other tools include the Neuropathic Pain Symptom Inventory, Neuropathic Pain Questionnaire, and painDETECT Questionnaire [117,118,119]. Quantitative sensory testing (QST) provides a detailed psychophysical method of quantitatively assessing neuropathic sensory abnormalities [120,121] using precise instruments such as von Frey filaments, Semmes-Weinstein monofilaments, weighted pinprick stimulators, Rydel-Seiffer tuning fork, pressure gauge devices, and thermal sensory testing devices.

Based on the findings of this systematic review, the following pathway should be regarded as an expert recommendation derived from the review, not as a protocol validated by the included evidence. Patients reporting donor-site pain or sensory symptoms after RFFF or FFF harvest can be screened at 3, 6, and 12 months, and thereafter as clinically indicated, using DN4 and a structured sensory examination comparing the donor site with the contralateral limb. Bedside assessment may include light-touch or monofilament testing, pinprick, cold/warm discrimination, dynamic mechanical allodynia, two-point discrimination when feasible, and Tinel’s test over the expected course of the SRN, LABCN, superficial peroneal nerve, and sural nerve [122,123,124]. DN4 ≥ 4, progressive sensory loss, allodynia/hyperalgesia, suspected neuroma, motor deficit, or persistent symptoms beyond 3 months should prompt referral to a pain or peripheral nerve specialist. Nerve conduction studies and EMG can help localize large-fiber lesions or concomitant motor involvement but may miss isolated small-fiber injury; QST can be considered when symptoms are discordant with bedside testing. Pharmacologic or surgical treatments, including neurolysis, neuroma excision, targeted muscle reinnervation, or regenerative peripheral nerve interface, should be individualized because the current donor-site literature does not validate a single treatment algorithm [114,125,126,127,128,129].

### 4.6. Limitations

This systematic review has several limitations. First, although exploratory random-effects quantitative summaries were added, high clinical and methodological heterogeneity means that pooled estimates should be interpreted cautiously and alongside medians, IQRs, and ranges. Second, outcome definitions were inconsistent; terms such as numbness, hypoesthesia, paresthesia, dysesthesia, sensory deficit, pain, and cold intolerance were not standardized and may not describe identical clinical phenomena across studies. Third, most studies were observational, retrospective, cross-sectional, or case-series designs, and formal risk-of-bias assessment showed predominantly moderate or high risk. Fourth, sensory outcomes were often secondary endpoints within broader donor-site morbidity studies, resulting in incomplete symptom characterization and limited use of validated neuropathic pain instruments. Fifth, exclusion of non-English studies may have introduced language bias. Sixth, ethical approval and informed consent reporting in the primary studies was inconsistent, especially in older publications. Finally, few studies reported detailed anatomical localization of sensory deficits, and none systematically combined neurological examination with nerve conduction studies/EMG, limiting causal attribution to specific nerve injuries.

## 5. Conclusions

This systematic review of 73 studies suggests that sensory abnormalities and chronic pain at RFFF and FFF donor sites are common but variably reported sources of postoperative morbidity. For RFFF, extractable sensory abnormality estimates had a median of 31.3% and an exploratory pooled prevalence of 34.4%, while general donor-site pain had a median of 12.0% and DN4-defined neuropathic pain was reported in 18% in the largest dedicated study. For FFF, extractable sensory abnormality estimates had a median of 21.4% and an exploratory pooled prevalence of 24.0%, while general donor-site pain had a median of 17.5% and DN4-defined neuropathic pain was reported in 21% in the only dedicated FFF study. These figures should be interpreted cautiously because heterogeneity was substantial.

The most consistent methodological finding is the gap between patient-reported symptoms and objective sensory assessment. Most studies relied on non-standardized patient-reported questionnaires, while objective sensory testing was applied inconsistently. No included study employed a comprehensive neurological examination combined with systematic nerve conduction studies or EMG to localize donor-site nerve injury after flap harvest.

Future prospective research would benefit from protocol-defined sensory outcomes, preoperative and contralateral comparison, standardized neuropathic pain screening (e.g., DN4, Neuropathic Pain Symptom Inventory, or painDETECT), structured neurological examination, and objective QST. Surgical studies should report nerve-preservation steps, donor-site closure techniques, and validated patient-reported outcomes so that the contribution of specific operative factors to long-term donor-site morbidity can be more reliably evaluated.

## Figures and Tables

**Table 1:** The characteristics of the included studies.

Study (Author, Year)	Study Design	LoE	Flap Type	Sample Size (n)	Follow-Up	Subjective Assessment	Objective Assessment	Key Sensory Outcomes
Bruin et al., 2023 [14]	Multicenter cross-sectional	3	RFFF	114	Variable	DN4 questionnaire	None	Neuropathic pain 18%; associated with tendon exposure (32%), poorer QoL (*p* < 0.001), impaired hand function (*p* < 0.001)
Lane et al., 2013 [15]	Cross-sectional	3	RFFF	45	Mean > 12 months	VAS (pain), patient-reported symptom questionnaire	Clinical sensory testing (light touch, sharp/blunt discrimination)	Pain VAS 7.8/10 (in pain reporters); hypoesthesia 42%; cold intolerance 16%; SRN territory deficits predominant
Lee et al., 2011 [16]	Cross-sectional	3	RFFF	19	Not specified	Patient-reported donor-site symptom questionnaire	Not specified	Pain 26%; hypoesthesia 89%—one of the highest rates reported in the RFFF literature
Loreti et al., 2008 [17]	Prospective cohort	2b	RFFF	17	Not specified	Patient questionnaires	Clinical sensory examination	Hypoesthesia 88%; sensory deficits concentrated in superficial radial nerve territory
De Bree et al., 2004 [18]	Cross-sectional	3	RFFF	37	≥6 months	Questionnaires (sensibility/cosmetics, forearm disabilities)	Wrist ROM, grip/pinch strength, temperature discrimination (digits I and V)	Hypoesthesia 46%; cold intolerance 16%; functional deficits in grip/pinch strength and wrist ROM; subjective sensibility complaints in majority
Grinsell et al., 2005 [19]	Retrospective comparative cohort	3	RFFF	51	Not specified	Patient-reported symptoms of radial nerve morbidity	Clinical assessment of radial nerve morbidity	Radial nerve morbidity 9% (venae comitantes group) vs. 18% (cephalic vein group); raising cephalic vein doubled incidence of sensory deficit in SRN territory
Novak et al., 2007 [20]	Comparative cross-sectional	3	RFFF	19 (RFFF arm)	Not specified	Patient-reported donor site morbidity questionnaire	None	Pain 37%; numbness 42%; hypoesthesia 11%; cold intolerance 26%; scar concerns 47%
Hekner et al., 2013 [21]	Comparative cross-sectional	3	RFFF	28 (RFFF group); 27 (ulnar group)	Not specified	Patient interview (sensory perception, grip/pinch strength, scar appearance)	Semmes-Weinstein monofilaments (pressure perception), cold perception testing (chloroethyl)	Reduced pressure perception and cold perception in RFFF donor hand; ulnar forearm flap group showed no significant sensory deficits; RFFF significantly worse for sensory outcomes
Schwarzer et al., 2016 [22]	Comparative prospective study (subfascial vs. suprafascial)	3	RFFF	50 (25 + 25)	3 months	Patient-reported donor-site complications	Clinical donor-site assessment, flap perfusion metrics	Sensory morbidity documented in both groups; no clear sensory superiority of subfascial over suprafascial technique
Pirlich et al., 2018 [23]	Cross-sectional	3	RFFF	39	Not specified	Pain-focused patient-reported assessment	Not specified	Donor-site pain 18%; neuropathic-type pain features identified; data also cited in Bruin et al. synthesis [8]
Kitano et al., 2023 [24]	Retrospective cohort	3	RFFF	39	Not specified	5-point symptom scale	SWM	Complete SRN preservation: subjective 1.40, objective 1.78; zero preservation: subjective 2.40, objective 3.18 (*p* = 0.019, *p* = 0.0035)
Timmons et al., 1986 [25]	Retrospective case series (two-centre)	4	RFFF	15	2 months to 2 years	Clinical and patient interview; cold intolerance	Clinical observation only; no formal sensory instrument	Reduced/abnormal sensation in radial nerve distribution: 47% (7/15); cold-induced symptoms: 27% (4/15); 3 of 4 cold-intolerance patients were female-to-male transsexuals on testosterone; no formal sensory testing
Boorman et al., 1987 [26]	Retrospective cohort	3	RFFF (25 free, 2 pedicle; 13 with bone)	27	2–36 months	Patient interview: spontaneous concerns; cold intolerance	Area of sensory loss; neuroma examination	Sensory loss in radial territory in 5/7 (71%) patients with dorsal paddle extension; only 1/27 (4%) overall; 2 neuromas (1 requiring surgery); no cold intolerance; sensory loss closely linked to dorsal paddle extension
Swanson et al., 1990 [27]	Retrospective case series	4	RFFF (fasciocutaneous and osteocutaneous; 13 with bone)	35 (30 seen at follow-up; functional data from 20)	Mean 10 months	Patient-reported satisfaction; questions about cold intolerance and numbness	No validated sensory instrument	Numbness in radial nerve distribution (dorsum of hand/first web space): 30% (9/30); no cold intolerance; no painful neuromas
Toschka et al., 2001 [28]	Retrospective cohort (with controls)	3	RFFF	35 patients; 15 non-operated controls	Mean 12.7 months	Questionnaire: aesthetic outcome (3-point scale); hand function (Quinnell test, 0–4); desire for corrective surgery	Stereognosis (12-object tactile identification); 2-point discrimination; proprioception; functional hand testing (13-item Quinnell series)	Hypoesthesia 77.1% (27/35); anesthesia 8.6% (3/35); hyperesthesia 8.6% (3/35); no impairment 5.7% (2/35); cold intolerance 31.4% (11/35); no clinically relevant differences in stereognosis, 2-PD, or proprioception vs. controls
Wax et al., 2001 [29]	Prospective RCT (scalpel vs. bipolar scissors)	1b	RFFF (fasciocutaneous)	40 (20 per arm)	6 months	Complication assessment (immediate and 6 months)	Clinical assessment of donor site complications	Radial nerve numbness 10% (2/20) in each group; no difference in sensory outcomes between arms
Sinha et al., 2002 [30]	Prospective, non-randomized cohort	2b	RFFF (fasciocutaneous, AlloDerm donor site closure)	52	Mean 14 months	Patient and physician assessment of wrist ROM (subjective comparison with contralateral side)	No objective sensory testing performed (explicitly stated by authors)	Sensory deficit 0% (0/52)—reported as absent based on absence of patient complaints and clinical observation only; no systematic sensory examination performed; seroma 10% as the only documented complication
Skoner et al., 2003 [31]	Retrospective case series	4	RFFF (fasciocutaneous; subfascial; SRN and paratenon preserved)	12	5–8 days postoperatively only	None (occupational therapist assessment only)	Sharp/dull sensation in radial, median, ulnar distributions (patient blinded); preoperative vs. postoperative within-extremity comparison	Diminished sharp sensation in anatomical snuffbox (SRN territory): 3/12 (25%); ulnar and median sensation intact in all; no LABCN/MABCN testing; short-term only—no recovery data
Sardesai et al., 2008 [32]	Retrospective cohort with internal controls	3	RFFF (fasciocutaneous; STSG from thigh)	16	Mean 817 days, SD 789 (~2.2 years)	Michigan Hand Outcomes Questionnaire (MHQ)—validated QoL instrument (70-item, 6 domains: function, ADL, work, pain, aesthetics, satisfaction)	No objective assessment	No dedicated sensory testing; MHQ pain domain significantly higher on operated side (+13.6 points, *p* = 0.045); MHQ hand function domain significantly worse (−10.9 points, *p* = 0.031); authors flag ‘alterations in index and thumb sensation’ as warranting further investigation
Faisal et al., 2013 [33]	RCT (RFFF vs. nasolabial flap)	1b	RFFF	25 (RFFF arm)	Mean 6 months (every 15 days)	Questionnaire: pigmentation, scar width, depression, wrist mobility, sensory abnormalities, pain, grip strength	Clinical examination; interincisal distance measurement	Loss of all sensations (donor hand) 12% (3/25); numbness 4% (1/25); itching 25% (5/25); cold intolerance 8% (2/25)
Riecke et al., 2015 [34]	Prospective panel study (longitudinal)	2b	RFFF	32 enrolled; 30 short-term; 20 long-term	Short-term: mean 95.6 days; Long-term: mean 2.2 years (535–1066 days)	DASH score, Mayo wrist score; subjective questionnaire (sensory limitations, appearance)	No objective sensory assessment	Persistent sensory hypoesthesia (thenar region) 20% (4/20) at long-term follow-up; chronic pain 0%; 90% patient satisfaction
Riecke et al., 2016 [35]	Prospective cohort	2b	RFFF	30	3 months postoperatively	DASH score, Mayo wrist score; questionnaire (appearance, sensitivity, subjective ROM)	No objective assessment	Sensory disturbance 50% (15/30): hypoesthesia 40% (12/30), dysesthesia 10% (3/30); no anesthesia; chronic pain 0%; 5 patients (17%) reported restricted subjective function; Mayo wrist score reduced 9.4 points (−12.9%)
Byun et al., 2016 [36]	Prospective cohort	2b	RFFF	10	6 months	Questionnaire: aesthetic satisfaction (VAS 0–10), pain, paresthesia, hand stiffness, reduced grip strength	Sensory disturbance area measurement (cm^2^)	Sensory disturbance area present in all 10 patients (mean 18.70 ± 4.39 cm^2^ at 6 months); moderately impaired hand sensation 20% (2/10) at 1 month, resolved by 6 months (0%); chronic pain 0%
Moreno-Sánchez et al., 2016 [37]	Prospective cohort (‘iberic graft’ closure technique)	2b	RFFF	100	Mean 36 months (range 6–78 months)	Patient history; binary assessment of dysesthesia presence	No validated sensory testing; ‘detailed history and thorough hand/wrist examination’	Temporal dysesthesia 2% (2/100) in SRN territory; definitive dysesthesia 0%; pain not assessed as a dedicated outcome; no functional deficit complaints
Wang et al., 2013 [38]	Retrospective comparative cohort (ALT vs. RFFF)	3	RFFF (fasciocutaneous)	44 (RFFF arm)	≥6 months postoperatively	Structured questionnaire	None	Donor site paresthesia 88.6% (39/44); donor site appearance dissatisfaction 86.4% (38/44); limb weakness 9.1% (4/44); no validated sensory instrument; no objective sensory testing; RFFF data are secondary outcomes from a study designed primarily to demonstrate ALT flap superiority for tongue reconstruction; no LABCN/MABCN distinction
Wang et al., 2018 [39]	Prospective cohort	2b	RFFF	31	Not specified	None specified	Comprehensive QST (thermal, SWM, pinprick, pressure algometry)	All QST parameters except PPT significantly different between donor and contralateral side; modified technique with ultrasonically activated shears showed better recovery
Chang et al., 2020 [40]	Prospective comparative	2b	RFFF	98 (50 RFFF + 48 ulnar)	Not specified	Questionnaires	SWM, ice application	No significant differences in light touch sensation or cold intolerance between RFFF and ulnar artery perforator flap groups
Deneuve et al., 2021 [41]	Cross-sectional	3	RFFF	191	Mean 34 months	5-point Likert questionnaire	None	Hypoesthesia 14.3%, paresthesia 12.2%, chronic pain 13.8%; no sex differences; sensory disturbances did not negatively impact QoL
Thiem et al., 2022 [42]	Randomized controlled trial	1b	RFFF	30 (15 RFFF + 15 UFFF)	Long-term	Likert scale, DASH score	Grip/pinch strength, hyperspectral imaging	Hypoesthesia: UFFF 47% vs. RFFF 27%; sensory morbidity higher in UFFF
Hong 2024 [43]	Retrospective cohort	3	RFFF	67	Not specified	Patient questionnaires	Clinical examination	Paresthesia 13%, itching 10%, scar pain 10%; risk factors: hypertension, diabetes, age; acellular matrix reduced paresthesia
Richardson et al., 1997 [44]	Prospective longitudinal	2b	RFFF	86	12 months	Patient-reported outcomes	Clinical examination	Reduced sensation: 75% at 3 months, 50% at 6 months, 32% at 12 months
Wirthmann et al., 2014 [45]	Cross-sectional	3	RFFF	Not specified	Mean 24 months	Patient questionnaires	Not specified	Sensory deficits persisted in 54% at mean 24 months follow-up
Cigna et al., 2015 [46]	Prospective cohort	2b	RFFF	15	Long-term	Not specified	Two-point discrimination	Impaired 2-point discrimination in 33% at long-term follow-up
Kerawala & Martin 2006 [47]	Prospective cohort	2b	RFFF	50	Not specified	Patient interview	Clinical sensory examination	Documented sensory deficits in donor hand in superficial radial nerve territory
Clark et al., 2019 [48]	Randomized controlled trial	1b	RFFF	24	3 months	Michigan Hand Questionnaire (MHQ)	Grip strength, ROM, Semmes-Weinstein monofilaments	No differences in sensation between NPD and SPD groups; improved self-reported hand function at 7 days with NPD (*p* = 0.016); improved flexion at 7 days (*p* = 0.031); no differences retained at 1–3 months
Jaquet et al., 2012 [49]	Case-control study	3	RFFF	44	≥6 months	VAS aesthetics/function, Michigan Hand Outcomes Questionnaire	Semmes-Weinstein monofilaments, pinch/grip dynamometry, wrist mobility	Significantly higher monofilament thresholds on operated vs. non-operated arms; values within normal ranges; UBTF closure showed no significant benefit over STSG for sensation
De Lange et al., 2025 [50]	Multicenter cross-sectional	3	FFF	150 (82 completed)	Variable	DN4, VAS, LEFS, EQ-5D-5L	None (questionnaire only)	Neuropathic pain 21% (DN4 ≥ 4), leg pain 27%, VAS median 5.0; hypoesthesia 49%, paresthesia 28%, itching 23%, stinging 23%, burning 18%, cold intolerance 17%, electrical shocks 12%
Pototschnig et al., 2013 [51]	Retrospective cohort	3	FFF	104	Not specified	Patient questionnaires	Not specified	Moderate pain in 8.7% of patients
Bartaire et al., 2012 [52]	Cross-sectional	3	FFF	23	Not specified	Patient questionnaires/interview	Tactile sensitivity, 2-PD, pain sensation testing	23% pain requiring analgesics; objective: preserved tactile sensitivity, normal 2-PD, intact pain sensation. Dissociation subjective vs. objective
Attia et al., 2020 [53]	Retrospective cohort	3	FFF	68	Not specified	Questionnaires, VAS	Not specified	Paresthesia 7.3%, persistent pain 4.4%, mean pain score 3.2/10, minimal QoL limitations
Xu et al., 2017 [54]	Prospective cohort	2b	FFF	30	6 months	POSAS, patient complaints	Isokinetic dynamometer, EMG, gait analysis (Footscan)	Lateral calf numbness 73.33%, chronic pain 6.67%, cold intolerance 6.67%; decreased ankle plantar/dorsiflexion work
Maben et al., 2021 [55]	Prospective cohort	2b	FFF	20	6 months	PES questionnaire	None	1 patient occasional numbness, 1 mild pain with ambulation; 45% pain-free at 6 months
Di Giuli et al., 2019 [56]	Prospective longitudinal	2b	FFF	14	Longitudinal	WOMAC, PES, clinical questioning	Gait analysis (SMART-E optoelectronic system), clinical sensory exam	21% activity-related pain, 1 patient pain at rest, 71% no residual alterations; 6% reduction in double-support phase
Ali et al., 2021 [57]	Prospective observational	2b	FFF	361	Variable	Patient questionnaires	AOFAS score	Early sensory deficit 5.26%; chronic pain 8.03%, ankle instability 6.64%, gait abnormality 8.76%, claw toe 9.14%; AOFAS 88.45%
Celotti et al., 2020 [58]	Retrospective case series	4	FFF	18	Not specified	Clinical interview	Clinical exam, radiological assessment	Cold intolerance 61.11%, motor weakness 55.56%, gait disorders 50%, sensory deficit 50%
Lee et al., 2023 [59]	Cross-sectional	3	FFF	39	Long-term	Questionnaires (5-category)	Medical chart review	Pain 10.3%, sensory disturbance 23.1%
Rashid et al., 2025 [60]	Retrospective study	3	Both	16 (10 RFFF, 6 FFF)	Not specified	Patient satisfaction	Clinical examination	RFFF: SRN sensory deficits 70%, cold intolerance 40%; FFF: cold intolerance 40%, leg swelling 40%, no foot drop
Cheon et al., 2025 [61]	Comparative (retrospective)	3	Both	Multiple flap types	Acute (24 h)	VAS, PCA monitoring	PCA consumption	FFF: relatively elevated pain 12–24 h postop; no significant differences in overall analgesia requirements between flap types
Hidalgo, 1989 [62]	Prospective cohort	2b	FFF (osseous and osteocutaneous)	12	Short-term only	Clinical observation and surgeon assessment of donor site morbidity	Clinical examination; no standardized sensory testing performed	No long-term donor site sensory morbidity documented in any patient (0%); no peroneal nerve sensory or motor deficit reported; no pain assessment performed; no neuropathic pain screening
Anthony et al., 1995 [63]	Prospective cohort	2b	FFF (osteocutaneous)	29 patients (30 flaps); 11 underwent isokinetic testing	Mean 7.3 months	Questionnaire: ankle/knee stiffness, pain, cold intolerance, numbness, weakness, paresthesias	Physical examination of peroneal nerve sensory and motor function standardized sensory mapping instrument	Superficial peroneal nerve sensory loss: 24% (7/29) at initial examination—completely resolved in all within 3 months; mild occasional donor-site discomfort: 28% (8/29); cold intolerance: 0%; no patient perceived objective deficits as functionally limiting
Hidalgo et al., 1995 [64]	Retrospective cohort with long-term follow-up	3	FFF (osseous 38%; osteocutaneous 62%; mandibular reconstruction only)	60 total; 40 available for long-term follow-up	Mean 39.2 months for disease-free survivors	Patient questionnaire and physical examination; static (pain, edema, paresthesias) and dynamic aspects assessed	Physical examination of ambulation; no standardized sensory instrument; no formal bilateral neurological comparison protocol described	15/40 (37.5%) had donor site symptoms: hypoesthesia 7 (17.5%), low-grade pain 7 (17.5%); 5 patients had two concurrent symptoms; 2 patients had limitations with running; all 40 ambulatory normally without assistive devices; hypoesthesia involved superficial or deep peroneal nerve distribution
Ferri et al., 1997 [65]	Retrospective cohort	3	FFF	29	Not specified (retrospective)	Clinical assessment of functional sequelae	No neurological clinical exam or sensory testing performed	No functional sequelae at donor site in any patient; one patient with residual leg pain for 3 months (transient); all patients ambulatory without limping by 1 month
Shpitzer et al., 1997 [66]	Prospective cohort	2b	FFF	47 patients (50 flaps); 41 donor sites available for long-term follow-up	Mean 17 months (range 4–39 months)	Patient questionnaire: pain, oedema, great toe weakness, ankle instability/stiffness; ability to engage in daily and recreational activities	Physical examination by same examiner sensation over medial and lateral borders of leg (deep and superficial peroneal and sural nerve territories) assessed by comparison with unoperated leg	No sensory loss reported by any patient (0%); 27% (11/41) had late donor site morbidity (all mild): ankle instability/stiffness 7%, donor site pain 2%, oedema 5%; no cold intolerance; all patients fully ambulatory
Shpitzer et al., 1999 [67]	Retrospective comparative cohort (FFF vs. iliac crest flap; partially overlapping with Shpitzer 1997 dataset [66])	3	FFF	58 FFF patients (62 flaps); 48 available for long-term follow-up	FFF group: mean 15 months (range 4–39 months)	Chart review of donor site complications; patient/surgeon functional assessment; no validated sensory questionnaire administered	Physical examination (same approach as Shpitzer 1997 [66]); no standardized sensory testing	Late FFF donor site morbidity: ankle stiffness/instability 3, donor site pain 1, oedema 2; no sensory deficit data reported for FFF group; data substantially overlaps with Shpitzer 1997 [66]; primary study aim was recipient-site functional comparison, not donor-site sensory characterisation
Babovic et al., 2000 [68]	Retrospective cohort	3	FFF	100 consecutive patients	Mean 17.42 months (range 3–60 months)	Patient-reported prolonged pain, limitations in ambulation; documentation at postoperative visits	Postoperative physical examination data reviewed from records; physical medicine and rehabilitation services evaluation for every patient; hypoesthesia over lateral lower leg evaluated; no standardized sensory instrument; two-point discrimination not performed; no bilateral sensory comparisons	Foot numbness (hypoesthesia, lateral lower leg/dorsum of foot) 10% (10/100); prolonged pharmacologic pain control beyond 6 weeks in 19% (19/100) with no detectable clinical complication; limited ambulatory distance (<1000 m) 27.3% (15/55 analysed); difficulty walking stairs 10.9% (6/55); most symptoms mild and not interfering with daily life
Shindo et al., 2000 [69]	Retrospective cohort with patient questionnaire	3	FFF (osteocutaneous; head and neck including tumour resection, trauma, osteoradionecrosis)	53 (27 available for long-term functional evaluation > 3 months)	Variable; 27 patients with >3 months follow-up for functional evaluation	Patient questionnaire: chronic pain (1–10), temperature intolerance, numbness/paresthesia, ADL effect scored 1 (minimal)–10 (debilitating)	Sensory and motor evaluation of donor leg; clinical examination of weakness and sensory deficits; no standardized neurological instrument; no validated sensory testing	Among 27 patients with long-term follow-up: sensory disturbance (numbness/paresthesia) 12/27 (44.4%) overall—Group 1 (major complications) 3/4 (75%), Group 2 (minor complications) 1/9 (11%), Group 3 (no complications) 8/14 (57%); chronic pain 8/27 (29.6%); primary aim was wound closure method comparison—sensory outcomes incidental; heavy smokers had significantly higher complication rate (*p* = 0.047)
Zimmermann et al., 2001 [70]	Prospective cohort	2b	FFF (osseous 38.1%; osteocutaneous 40.5%; osteomuscular 7.1%; osteomyocutaneous 14.3%)	42 evaluated (from 86 harvested; 36 died, 8 lost to follow-up)	Mean 35 months (range 1–99 months, SD ± 29 months)	Standardized patient interview: discomfort at rest and in motion, recurrent pain (NRS 0–10), temperature difference, sensory abnormalities; motor limitations, daily life restrictions, aesthetic satisfaction	Physical examination vs. contralateral limb: sensory evaluation in specified calf regions using cotton swab and standardized pressure probe testing; area of deficit recorded in cm^2^	Objective sensory deficits: 76.3% (29/38); subjective sensory deficits: 47.4% (18/38); touch hypoesthesia: 60.5% (23/38); pressure hypoesthesia: 50% (19/38); anesthesia: 36.8% (14/38); mean sensory deficit area: touch 122 cm^2^, pressure 129 cm^2^; chronic recurrent pain: 23.7% (9/38), mean NRS 4.4 ± 1.5; discomfort at rest: 23.7%; discomfort in motion: 21.1%; 5 patients with obvious gait abnormalities
Muñoz Guerra et al., 2001 [71]	Retrospective cohort	3	FFF (osteomuscular, with skin paddle in 24/26; ‘double barrel’ in 6 cases)	26	3–32 months	Clinical evaluation of donor site complications; no validated questionnaire administered	Clinical examination; no neurological or sensory testing performed	Pain (transitory) 38.46% (10/26); peroneal sensory loss (transitory) 26.92% (7/26); all complications described as transitory; no long-term functional complications; ambulation pain-free by 3 months; zero long-term rate reflects absence of systematic follow-up measurement
Hidalgo & Pusic 2002 [72]	Retrospective cohort (single surgeon series, survey)	3	FFF (19/20 fibula; 1/20 scapula)	20 (from 84 identified; 34 alive; 20 participated; response rate 67%)	Mean 11 years (range 10–14 years)	Survey combining validated and study-specific questions; patient interview in person (14) or by telephone (6)	Clinical oral examination (14 patients); no dedicated donor site sensory testing performed, donor site sensory morbidity assessed incidentally	No significant long-term disability related to donor site; 3/20 (15%) with mild intermittent leg weakness or pain; 1/20 (5%) with symptoms severe enough to curtail jogging
Bodde et al., 2003 [73]	Prospective cohort with age-matched controls	2b	FFF (all included skin paddle)	10 patients; 6 age-matched healthy controls	6–87 months (median 26 months)	Patient questionnaire: pain (VAS), walking ability, daily activity restrictions, Enneking functional evaluation system	No objective asssessment of sensory deficit	Pain 60% (6/10); sensory disturbances (dysesthesia/paresthesia/numbness) 50% (5/10); only 40% had no complaints
Garrett et al., 2006 [74]	Retrospective case series	4	FFF (osteomyocutaneous)	14	Variable (cases from 1997–2003; specific follow-up duration not reported)	Ankle-hindfoot scale (AOFAS; 100-point: pain, function, ROM, stability, alignment); patient-reported pain and ambulation capacity	No dedicated sensory or neurological testing	Mean ankle-hindfoot score 84.82/100 (range 55–100); pain in 6/14 (42.9%)—5 mild/occasional, 1 moderate (attributed to pre-existing peripheral vascular disease); primary focus on ankle biomechanics and radiographic stability, not sensory outcomes
Farhadi et al., 2007 [75]	Retrospective cohort	3	FFF	10	Mean 32.3 months	Standardized questionnaire; morbidity point-evaluation system (16-point composite); AOFAS hindfoot score; patient-reported pain and instability	Independent neurologist performed a clinical exam; no standardized sensory instrument	3/10 (30%) patients reported mild donor site pain, 1/10 (10%) patient reported distressing pain; 1/10 patients reported numbness, 5/10 (50%) patients reported sensibility to weather. No dedicated sensory testing performed; no validated neuropathic pain instrument; small sample limits generalisability
Lee et al., 2008 [76]	Prospective cohort (pre- and post-operative gait analysis)	2b	FFF	20 (pre-op and 1-month post-op); 12 consented to 3-month post-op analysis	1 month and 3 months postoperatively	Patient-reported pain	No objective assessment	All 20 reported mild-moderate donor site pain at 1 month; all negative at 3 months (except ankle pain with hard exercise only); 12/20 attrition at 3 months reduces reliability of 3-month data
González-García et al., 2008 [77]	Retrospective cohort	3	FFF (osteocutaneous 90.4%; osseous 9.6%)	42	Variable; range 0–60 months per patient; mean follow-up not reported	Surgeon/patient assessment of functional and aesthetic outcomes; donor site complications from medical charts; no validated sensory questionnaire administered	Clinical examination and chart-based complication review; no standardized sensory assessment of the donor leg performed	Donor site complications in 5/42 (11.9%): pain noted in 3 individual patients
Papadopulos et al., 2008 [78]	Retrospective cohort	3	FFF osteofasciocutaneous	23	Mean 1.3 years (range 1–2 years)	Patient questionnaire: pain, swelling, intolerance of weather variation, limitations in working or private life	Clinical examination no standardized sensory instrument; no neurological testing protocol described	Donor-site morbidity described as ‘moderate’; 3/23 (13%) patients reported disturbance of sensation of the lateral lower leg and foot; 3/23 (13%) patients reported lower leg pain after overexertion; 1/23 (4%) reported intolerance of weather variations; specific sensory deficit rates not reported as separate figures.
Chou et al., 2009 [79]	Retrospective case-control	4	FFF (osteoseptocutaneous)	11 patients; 10 age-matched healthy controls	Mean 27.4 months (range 10–68 months)	Patient questionnaire: pain, great toe/leg weakness, sensory disturbances, ankle/knee instability	No objective sensory testing	Sural nerve distribution sensory impairment 9.1% (1/11); pain with prolonged standing 9.1% (1/11)
Sieg et al., 2010 [80]	Cross-sectional	3	FFF (osseous 37%, osteocutaneous 48%, osseomyocutaneous 10%, osseomuscular 5%)	57 patients (62 donor regions), from 165 total reconstructions	2–167 months (mean 45 months, median 27 months)	Patient-reported symptoms; Kitaoka ankle-hindfoot score (100-point scale incorporating pain, function, and alignment)	Standardized clinical examination by same examiner: neurological assessment of sural, superficial peroneal, and deep peroneal nerve territories (dysesthesia)	Dysesthesia 48% (30/62): sural nerve 26% (16), superficial peroneal nerve 31% (19), deep peroneal nerve 32% (20); Kitaoka score: mean 87, median 93; excellent/good results 84% (52/62); poor results 10% (6/62); wound healing complications 21%; no correlation between any of 15 risk factors and functional impairment
López-Arcas et al., 2010 [81]	Retrospective cohort	3	FFF (osteocutaneous/osteomyocutaneous)	117	Mean 46 months (range 3–97 months)	Surgeon/patient assessment of functional and aesthetic outcomes	Clinical examination; no standardized sensory testing of the donor leg performed	Calf paresthesias: 21.4% (25/117)—most frequent donor site complication; peroneal nerve damage: 1.7% (2/117); >85% of patients had essentially normal leg function; pain not assessed as a dedicated outcome variable; no severity grading of paresthesias
Wolff et al., 2011 [82]	Prospective cohort	2b	FFF (osteocutaneous 94%; osseous 6%; oral reconstruction—mandible 88%, maxilla 12%)	66 flaps raised; 63 with >3-month donor site follow-up	Mean 17.5 months (range 3–32 months)	Patient-reported pain, dysesthesia, cold intolerance; assessment during routine follow-up	Clinical inspection of donor site at follow-up; no standardized neurological examination protocol; no bilateral sensory comparison	Transient sensory deficits (dysesthesia, cold intolerance, or initial moderate pain—grouped as single category) 18.2% (12/66)—all completely resolved by 6 months; permanent numbness around lateral ankle 3.0% (2/66) due to deliberate sural nerve sacrifice during large skin paddle elevation; permanent pain on stairs/long walks 1.5% (1/66); pain, dysesthesia and cold intolerance grouped together, precluding separation of sensory from pain outcomes
Ling et al., 2013 [83]	Retrospective comparative cohort (FFF vs. DCIA flap)	3	FFF (19 patients) vs. DCIA (15 patients)	34 (19 FFF, 15 DCIA flap)	Variable; evaluation period May 2010–February 2011; flaps performed 1998–2009	Standardized patient interview: scar, functional loss, wound healing, iatrogenic complications, pain	Physical examination: sensory deficit mapping with sharp dental probe, no validated sensory instrument	Fibula group: objective sensory deficit 0/19 (0%); DCIA group: neurosensory deficit in lateral thigh 4/15 (27%)—significant difference (*p* ≤ 0.05); apparent zero sensory deficit rate likely attributable to qualitative sharp-probe method which may underdetect subtle neuropathic changes
Rendenbach et al., 2016 [84]	Prospective cohort	2b	FFF (mandibular reconstruction)	27	Mean 8 months (pre- and postoperative comparison)	Patient questionnaire (AOFAS rating system): load-dependent pain, sensory deficits (paresthesia, anesthesia, hypoesthesia)	No objective sensory assessment	Sensory deficits (paresthesia, anesthesia, hypoesthesia): 13/27 (48.1%); load-dependent pain: 14/27 (51.4%)
Feuvrier et al., 2016 [85]	Prospective cohort with healthy age-matched controls	2b	FFF	11 FFF patients; 11 age-matched healthy controls	Mean 28 months (range 5–104 months)	Kitaoka Score, Point Evaluation System (PES) score; non-structured clinical questioning on pain, paresthesia, and ankle instability	Clinical and neurological examination of legs; no standardized sensory instrument; comparison with healthy controls	Sensory disorder (fibular nerve territory): 5/11 (45.5%)—paresthesia in 3/5; pain: 8/11 (72.7%)
Akashi et al., 2016 [86]	Retrospective cohort	3	FFF osteocutaneous	35 (24 primary closure, 11 skin graft)	Median 17 months (range 4–54 months)	Simple 4-item questionnaire at latest visit	None—late complaints assessed by questionnaire only; no neurological examination; no sensory testing performed	Late donor site complaints in 12/35 (34.3%): pain 7/35 (20.0%), numbness 1/35 (2.9%); complaints attributed to paresthesia around donor site, not peroneal nerve paralysis; early donor site morbidity 2/35 (5.7%); no significant difference between primary closure and skin graft groups in late complaints or early morbidity; no risk factors

**Table 2:** Quantitative summary of extractable donor-site sensory and pain outcomes.

Outcome	Studies (k)	Median Prevalence (IQR; Range)	Exploratory Random-Effects Pooled Prevalence (95% CI)	I2
RFFF sensory abnormality	24	31.3% (13.1–55.0; 0–100)	34.4% (23.6–47.1)	88.4%
RFFF general pain	8	12.0% (0–20.0; 0–36.8)	16.0% (9.9–24.8)	56.6%
RFFF neuropathic pain	2	18.2% (18.1–18.3; 17.9–18.4)	18.7% (13.3–25.6)	0%
RFFF cold intolerance	9	16.2% (8.0–26.7; 0–40.0)	20.1% (13.0–29.9)	50.2%
FFF sensory abnormality	27	21.4% (8.2–46.8; 0–76.3)	24.0% (16.5–33.6)	88.6%
FFF general pain	29	17.5% (7.1–27.6; 0–72.7)	18.8% (13.9–24.9)	77.7%
FFF neuropathic pain	1	20.7% (single study)	Not pooled; 21% in one dedicated DN4 study	Not applicable
FFF cold intolerance	7	6.7% (2.2–33.5; 0–61.1)	15.0% (5.4–35.2)	82.0%

Note: IQR, interquartile range; RFFF, radial forearm free flap; FFF, fibula free flap; DN4, Douleur Neuropathique en 4 Questions. Pooled estimates are exploratory because clinical and methodological heterogeneity was high.

## Data Availability

Data extracted for the qualitative and quantitative summaries are available in the manuscript and Supplementary File S1.

## Supplementary Materials

The following supporting information can be downloaded at: https://www.mdpi.com/article/10.3390/medicina62081581/s1, Methods S1: Database-Specific Search Strategies; Table S1: PRISMA 2020 Checklist; Table S2: Individual Study Risk-of-Bias Assessment; Table S3: Donor-Site Closure Technique or Closure-Related Context; Table S4: Sensitivity Analysis Summary; Table S5: Comparison with Prior Systematic Reviews.
