# Supplementary material for: Sensory Abnormalities and Pain in the Donor Sites of Radial Forearm and Fibula Free Flaps—A Systematic Review
Source: Medicina (Kaunas). 2026 Aug 18;62(8):1581. doi: 10.3390/medicina62081581 (PMC13515868; doi:10.3390/medicina62081581)
Supplement: Supplementary file 1 [file medicina-62-01581-s001.zip › medicina-4380612-supplementary.pdf]

Supplementary Methods S1. Database-Specific Search Strategies

All searches were run from database inception to 1 October 2025. No date filter was applied. The final screening stage excluded non-English publications, case reports, and case series with fewer than 10 patients. Prior systematic reviews were used for citation tracking and contextual comparison but were not included as units of analysis.

Database/source	Search string and retrieval procedure
PubMed/MEDLINE	(("radial forearm free flap"[tiab] OR RFFF[tiab] OR "forearm flap"[tiab] OR "fibula free flap"[tiab] OR "free fibula flap"[tiab] OR FFF[tiab] OR "free tissue transfer"[tiab]) AND ("donor site"[tiab] OR "donor-site"[tiab] OR "donor site morbidity"[tiab]) AND (sensory[tiab] OR "sensory deficit"[tiab] OR "sensory disturbance"[tiab] OR hypoesthesia[tiab] OR hypesthesia[tiab] OR numbness[tiab] OR paresthesia[tiab] OR dysesthesia[tiab] OR pain[tiab] OR "neuropathic pain"[tiab] OR neuropathy[tiab] OR "cold intolerance"[tiab])).
Scopus	TITLE-ABS-KEY(("radial forearm free flap" OR RFFF OR "fibula free flap" OR "free fibula flap" OR FFF OR "free tissue transfer") AND ("donor site" OR "donor-site" OR "donor site morbidity") AND (sensory OR "sensory deficit" OR "sensory disturbance" OR hypoesthesia OR hypesthesia OR numbness OR paresthesia OR dysesthesia OR pain OR "neuropathic pain" OR neuropathy OR "cold intolerance")).
Web of Science Core Collection	TS=(("radial forearm free flap" OR RFFF OR "fibula free flap" OR "free fibula flap" OR FFF OR "free tissue transfer") AND ("donor site" OR "donor-site" OR "donor site morbidity") AND (sensory OR "sensory deficit" OR "sensory disturbance" OR hypoesthesia OR hypesthesia OR numbness OR paresthesia OR dysesthesia OR pain OR "neuropathic pain" OR neuropathy OR "cold intolerance")).
Cochrane Library	("radial forearm free flap" OR RFFF OR "fibula free flap" OR "free fibula flap" OR FFF OR "free tissue transfer") in Title Abstract Keyword AND ("donor site" OR "donor-site" OR "donor site morbidity") AND (sensory OR "sensory deficit" OR hypoesthesia OR numbness OR paresthesia OR dysesthesia OR pain OR "neuropathic pain" OR "cold intolerance").
Google Scholar	Four simplified queries were run: (1) "radial forearm free flap" "donor site" sensory pain; (2) "radial forearm free flap" neuropathic pain donor site; (3) "fibula free flap" donor site sensory pain; (4) "fibula free flap" neuropathic pain donor site. The first 200 results per query, sorted by relevance, were screened.
Gray literature and citation tracking	Reference lists of previous systematic reviews and included primary studies were screened. Indexed theses/dissertations and clinical-trial registry records were checked when identifiable through database or Google Scholar searches. Conference abstracts without full outcome data were excluded.

Supplementary Table S1. PRISMA 2020 Checklist

Section	Item	Checklist element	Location
Title	1	Identify the report as a systematic review.	Title page
Abstract	2	Provide a structured abstract including objectives, methods, results, and limitations.	Abstract
Rationale	3	Describe the rationale for the review in the context of existing knowledge.	Introduction
Objectives	4	Provide an explicit statement of the review questions.	Introduction/Objectives
Eligibility criteria	5	Specify inclusion and exclusion criteria.	Methods 2.4
Information sources	6	Specify all databases, registers, websites, organizations, and other sources searched.	Methods 2.2 and Supplementary Methods S1
Search strategy	7	Present full search strategies for all databases.	Supplementary Methods S1
Selection process	8	Specify methods used to decide whether studies met inclusion criteria.	Methods 2.5
Data collection process	9	Specify methods used to collect data from reports.	Methods 2.6
Data items	10	List and define all outcomes and variables sought.	Methods 2.6-2.7
Risk of bias in individual studies	11	Specify methods used to assess risk of bias.	Methods 2.8 and Supplementary Table S2
Effect measures	12	Specify effect measures.	Methods 2.9
Synthesis methods	13	Describe eligibility for synthesis, summary methods, and heterogeneity assessment.	Methods 2.9
Reporting bias assessment	14	Describe methods for reporting bias assessment.	Limitations
Certainty assessment	15	Describe methods for assessing certainty.	Methods 2.8 and Results 3.6-3.7
Study selection results	16	Report numbers screened, included, and excluded.	Results 3.1 and Figure 1
Study characteristics	17	Cite each included study and present characteristics.	Results 3.2 and Table 1
Risk of bias results	18	Present risk-of-bias assessments.	Results 3.7 and Supplementary Table S2
Results of individual studies	19	Present results for each included study.	Table 1

Results of syntheses	20	Present results of each synthesis.	Results 3.3-3.8 and Table 2
Reporting biases	21	Present assessments of reporting biases.	Limitations
Certainty of evidence	22	Present certainty assessment.	Results 3.6-3.7 and Discussion
Discussion	23	Provide general interpretation, limitations, and implications.	Discussion
Registration and protocol	24a	Provide registration information.	Methods 2.1
Protocol availability	24b	Indicate where protocol can be accessed.	Methods 2.1
Support	25	Describe sources of financial or non-financial support.	Funding
Competing interests	26	Declare conflicts of interest.	Conflicts of Interest
Availability of data/code	27	Report availability of extracted data and analytic code.	Data Availability Statement and Supplementary File S1

Supplementary Table S2. Individual Study Risk-of-Bias Assessment

Overall judgments: low risk=9, moderate risk=42, high risk=22. Judgments reflect risk of bias for donor-site sensory/pain outcomes, not necessarily the global quality of each surgical study.

Study	Design / LoE	Tool used	Overall judgment	Main limitations informing judgment
Bruin et al., 2023 [14]	Multicenter cross-sectional / 3	JBI analytical cross-sectional checklist	Low	limited or no objective sensory testing; follow-up incompletely standardized; observational design
Lane et al., 2013 [15]	Cross-sectional / 3	JBI analytical cross-sectional checklist	Low	observational design
Lee et al., 2011 [16]	Cross-sectional / 3	JBI analytical cross-sectional checklist	High	limited or no objective sensory testing; follow-up incompletely standardized; small cohort
Loreti et al., 2008 [17]	Prospective cohort / 2b	JBI cohort checklist	Moderate	follow-up incompletely standardized; small cohort
De Bree et al., 2004 [18]	Cross-sectional / 3	JBI analytical cross-sectional checklist	Low	observational design
Grinsell et al., 2005 [19]	Retrospective comparative cohort / 3	JBI cohort checklist	Moderate	follow-up incompletely standardized; observational design
Novak et al., 2007 [20]	Comparative cross-sectional / 3	JBI analytical cross-sectional checklist	Moderate	limited or no objective sensory testing; follow-up incompletely standardized; small cohort
Hekner et al., 2013 [21]	Comparative cross-sectional / 3	JBI analytical cross-sectional checklist	High	follow-up incompletely standardized; observational design
Schwarzer et al., 2016 [22]	Comparative prospective study (subfascial vs suprafascial) / 3	JBI cohort checklist	Moderate	comparatively stronger design/reporting for this evidence base
Pirlich et al., 2018 [23]	Cross-sectional / 3	JBI analytical cross-sectional checklist	Moderate	limited or no objective sensory testing; follow-up incompletely standardized; observational design
Kitano et al., 2023 [24]	Retrospective cohort / 3	JBI cohort checklist	Moderate	follow-up incompletely standardized; observational design
Timmons et al., 1986 [25]	Retrospective case series (two-centre) / 4	JBI case-series checklist	High	small cohort; observational design
Boorman et al., 1987 [26]	Retrospective cohort / 3	JBI cohort checklist	High	observational design
Swanson et al., 1990 [27]	Retrospective case series / 4	JBI case-series checklist	High	observational design
Toschka et al., 2001 [28]	Retrospective cohort (with controls) / 3	JBI cohort checklist	Moderate	observational design
Wax et al., 2001 [29]	Prospective RCT (scalpel vs. bipolar scissors) / 1b	JBI RCT checklist	Moderate	comparatively stronger design/reporting for this evidence base
Sinha et al., 2002 [30]	Prospective, non-randomized cohort / 2b	JBI RCT checklist	Moderate	limited or no objective sensory testing
Skoner et al., 2003 [31]	Retrospective case series / 4	JBI case-series checklist	High	small cohort; observational design
Sardesai et al., 2008 [32]	Retrospective cohort with internal controls / 3	JBI cohort checklist	Moderate	limited or no objective sensory testing; small cohort; observational design
Faisal et al., 2013 [33]	RCT (RFFF vs. nasolabial flap) / 1b	JBI RCT checklist	Moderate	comparatively stronger design/reporting for this evidence base
Riecke et al., 2015 [34]	Prospective panel study (longitudinal) / 2b	JBI cohort checklist	Moderate	limited or no objective sensory testing
Riecke et al., 2016 [35]	Prospective cohort / 2b	JBI cohort checklist	Moderate	limited or no objective sensory testing
Byun et al., 2016 [36]	Prospective cohort / 2b	JBI cohort checklist	High	small cohort
Moreno-Sánchez et al., 2016 [37]	Prospective cohort ('iberic graft' closure technique) / 2b	JBI cohort checklist	Moderate	comparatively stronger design/reporting for this evidence base
Wang et al., 2013 [38]	Retrospective comparative cohort (ALT vs. RFFF) / 3	JBI cohort checklist	Moderate	limited or no objective sensory testing; observational design; sensory outcome not always primary
Wang et al., 2019 [39]	Prospective cohort / 2b	JBI cohort checklist	Low	follow-up incompletely standardized
Chang et al., 2020 [40]	Prospective comparative / 2b	JBI cohort checklist	Moderate	follow-up incompletely standardized
Deneuve et al., 2021 [41]	Cross-sectional / 3	JBI analytical cross-sectional checklist	Low	limited or no objective sensory testing; observational design
Thiem et al., 2022 [42]	Randomized controlled trial / 1b	JBI RCT checklist	Moderate	comparatively stronger design/reporting for this evidence base
Hong 2024 [43]	Retrospective cohort / 3	JBI cohort checklist	Moderate	follow-up incompletely standardized; observational design
Richardson et al., 1997 [44]	Prospective longitudinal / 2b	JBI cohort checklist	Low	comparatively stronger design/reporting for this evidence base
Wirthmann et al., 2014 [45]	Cross-sectional / 3	JBI analytical cross-sectional checklist	Moderate	limited or no objective sensory testing; observational design
Cigna et al., 2015 [46]	Prospective cohort / 2b	JBI cohort checklist	Moderate	small cohort
Kerawala & Martin 2006 [47]	Prospective cohort / 2b	JBI cohort checklist	Moderate	follow-up incompletely standardized

Clark et al., 2019 [48]	Randomized controlled trial / 1b	JBI RCT checklist	Moderate	comparatively stronger design/reporting for this evidence base
Jaquet et al., 2012 [49]	Case-control study / 3	JBI analytical cross-sectional checklist	Moderate	comparatively stronger design/reporting for this evidence base
De Lange et al., 2025 [50]	Multicenter cross-sectional / 3	JBI analytical cross-sectional checklist	Low	limited or no objective sensory testing; follow-up incompletely standardized; observational design
Pototschnig et al., 2013 [51]	Retrospective cohort / 3	JBI cohort checklist	Moderate	limited or no objective sensory testing; follow-up incompletely standardized; observational design
Bartaire et al., 2012 [52]	Cross-sectional / 3	JBI analytical cross-sectional checklist	High	follow-up incompletely standardized; observational design
Attia et al., 2020 [53]	Retrospective cohort / 3	JBI cohort checklist	Moderate	limited or no objective sensory testing; follow-up incompletely standardized; observational design
Xu et al., 2017 [54]	Prospective cohort / 2b	JBI cohort checklist	Low	comparatively stronger design/reporting for this evidence base
Maben et al., 2021 [55]	Prospective cohort / 2b	JBI cohort checklist	High	limited or no objective sensory testing
Di Giuli et al., 2019 [56]	Prospective longitudinal / 2b	JBI cohort checklist	High	small cohort
Ali et al., 2021 [57]	Prospective observational / 2b	JBI cohort checklist	Low	follow-up incompletely standardized
Celotti et al., 2020 [58]	Retrospective case series / 4	JBI case-series checklist	High	follow-up incompletely standardized; small cohort; observational design
Lee et al., 2023 [59]	Cross-sectional / 3	JBI analytical cross-sectional checklist	Moderate	observational design
Rashid et al., 2025 [60]	Retrospective study / 3	JBI analytical cross-sectional checklist	High	follow-up incompletely standardized; small cohort; observational design
Cheon et al., 2025 [61]	Comparative (retrospective) / 3	JBI analytical cross-sectional checklist	High	observational design
Hidalgo, 1989 [62]	Prospective cohort / 2b	JBI cohort checklist	High	small cohort
Anthony et al., 1995 [63]	Prospective cohort / 2b	JBI cohort checklist	Moderate	comparatively stronger design/reporting for this evidence base
Hidalgo et al., 1995 [64]	Retrospective cohort with long-term follow-up / 3	JBI cohort checklist	Moderate	observational design
Ferri et al., 1997 [65]	Retrospective cohort / 3	JBI cohort checklist	High	follow-up incompletely standardized; observational design
Shpitzer et al., 1997 [66]	Prospective cohort / 2b	JBI cohort checklist	Moderate	comparatively stronger design/reporting for this evidence base
Shpitzer et al., 1999 [67]	Retrospective comparative cohort (FFF vs iliac crest flap; partially overlapping with Shpitzer 1997 dataset) / 3	JBI cohort checklist	High	observational design; sensory outcome not always primary
Babovic et al., 2000 [68]	Retrospective cohort / 3	JBI cohort checklist	Moderate	observational design
Shindo et al., 2000 [69]	Retrospective cohort with patient questionnaire / 3	JBI cohort checklist	Moderate	follow-up incompletely standardized; observational design; sensory outcome not always primary
Zimmermann et al., 2001 [70]	Prospective cohort / 2b	JBI cohort checklist	Moderate	comparatively stronger design/reporting for this evidence base
Muñoz Guerra et al., 2001 [71]	Retrospective cohort / 3	JBI cohort checklist	Moderate	observational design
Hidalgo & Pusic 2002 [72]	Retrospective cohort (single surgeon series, survey) / 3	JBI cohort checklist	High	observational design
Bodde et al., 2003 [73]	Prospective cohort with age-matched controls / 2b	JBI cohort checklist	High	limited or no objective sensory testing; small cohort
Garrett et al., 2006 [74]	Retrospective case series / 4	JBI case-series checklist	High	follow-up incompletely standardized; small cohort; observational design
Farhadi et al., 2007 [75]	Retrospective cohort / 3	JBI cohort checklist	High	small cohort; observational design
Lee et al., 2008 [76]	Prospective cohort (pre- and post-operative gait analysis) / 2b	JBI cohort checklist	Moderate	limited or no objective sensory testing
González-García et al., 2008 [77]	Retrospective cohort / 3	JBI cohort checklist	Moderate	follow-up incompletely standardized; observational design
Papadopulos et al., 2008 [78]	Retrospective cohort / 3	JBI cohort checklist	Moderate	observational design
Chou et al., 2009 [79]	Retrospective case-control / 4	JBI case-series checklist	High	limited or no objective sensory testing; small cohort; observational design
Sieg et al., 2010 [80]	Cross-sectional / 3	JBI analytical cross-sectional checklist	Moderate	observational design
López-Arcas et al., 2010 [81]	Retrospective cohort / 3	JBI cohort checklist	Moderate	observational design
Wolff et al., 2011 [82]	Prospective cohort / 2b	JBI cohort checklist	Moderate	comparatively stronger design/reporting for this evidence base
Ling et al., 2013 [83]	Retrospective comparative cohort (FFF vs DCIA flap) / 3	JBI cohort checklist	Moderate	follow-up incompletely standardized; observational design
Rendenbach et al., 2016 [84]	Prospective cohort / 2b	JBI cohort checklist	Moderate	limited or no objective sensory testing
Feuvrier et al., 2016 [85]	Prospective cohort with healthy age-matched controls / 2b	JBI cohort checklist	High	small cohort
Akashi et al., 2016 [86]	Retrospective cohort / 3	JBI cohort checklist	Moderate	limited or no objective sensory testing; observational design; sensory outcome not always primary

Supplementary Table S3. Donor-Site Closure Technique or Closure-Related Context

Study	Flap type	Closure/technique context extracted for revision
Bruin et al., 2023 [14]	RFFF	not reported or not sufficiently specified in extracted sensory-outcome table
Lane et al., 2013 [15]	RFFF	local hatchet flap for RFFF donor-site closure
Lee et al., 2011 [16]	RFFF	not reported or not sufficiently specified in extracted sensory-outcome table
Loreti et al., 2008 [17]	RFFF	not reported or not sufficiently specified in extracted sensory-outcome table
De Bree et al., 2004 [18]	RFFF	RFFF donor-site morbidity after fasciocutaneous radial forearm flap; closure details not uniformly extracted

Grinsell et al., 2005 [19]	RFFF	not reported or not sufficiently specified in extracted sensory-outcome table
Novak et al., 2007 [20]	RFFF	not reported or not sufficiently specified in extracted sensory-outcome table
Hekner et al., 2013 [21]	RFFF	not reported or not sufficiently specified in extracted sensory-outcome table
Schwarzer et al., 2016 [22]	RFFF	not reported or not sufficiently specified in extracted sensory-outcome table
Pirlich et al., 2018 [23]	RFFF	not reported or not sufficiently specified in extracted sensory-outcome table
Kitano et al., 2023 [24]	RFFF	not reported or not sufficiently specified in extracted sensory-outcome table
Timmons et al., 1986 [25]	RFFF	not reported or not sufficiently specified in extracted sensory-outcome table
Boorman et al., 1987 [26]	RFFF (25 free, 2 pedicle; 13 with bone)	not reported or not sufficiently specified in extracted sensory-outcome table
Swanson et al., 1990 [27]	RFFF (fasciocutaneous and osteocutaneous; 13 with bone)	not reported or not sufficiently specified in extracted sensory-outcome table
Toschka et al., 2001 [28]	RFFF	not reported or not sufficiently specified in extracted sensory-outcome table
Wax et al., 2001 [29]	RFFF (fasciocutaneous)	RFFF harvest using scalpel versus bipolar scissors
Sinha et al., 2002 [30]	RFFF (fasciocutaneous, AlloDerm donor site closure)	AlloDerm for RFFF donor-site coverage
Skoner et al., 2003 [31]	RFFF (fasciocutaneous; subfascial; SRN and paratenon preserved)	not reported or not sufficiently specified in extracted sensory-outcome table
Sardesai et al., 2008 [32]	RFFF (fasciocutaneous; STSG from thigh)	split-thickness skin graft from thigh
Faisal et al., 2013 [33]	RFFF	RFFF versus nasolabial flap comparison; donor-site symptoms recorded
Riecke et al., 2015 [34]	RFFF	local full-thickness skin graft closure
Riecke et al., 2016 [35]	RFFF	local full-thickness skin graft closure; biomechanical follow-up
Byun et al., 2016 [36]	RFFF	porcine collagen membrane versus conventional skin grafting
Moreno-Sánchez et al., 2016 [37]	RFFF	Iberic graft closure technique
Wang et al., 2013 [38]	RFFF (fasciocutaneous)	not reported or not sufficiently specified in extracted sensory-outcome table
Wang et al., 2019 [39]	RFFF	not reported or not sufficiently specified in extracted sensory-outcome table
Chang et al., 2020 [40]	RFFF	not reported or not sufficiently specified in extracted sensory-outcome table
Deneuve et al., 2021 [41]	RFFF	not reported or not sufficiently specified in extracted sensory-outcome table
Thiem et al., 2022 [42]	RFFF	not reported or not sufficiently specified in extracted sensory-outcome table
Hong 2024 [43]	RFFF	not reported or not sufficiently specified in extracted sensory-outcome table
Richardson et al., 1997 [44]	RFFF	not reported or not sufficiently specified in extracted sensory-outcome table
Wirthmann et al., 2014 [45]	RFFF	Integra coverage after RFFF elevation
Cigna et al., 2015 [46]	RFFF	not reported or not sufficiently specified in extracted sensory-outcome table
Kerawala & Martin 2006 [47]	RFFF	not reported or not sufficiently specified in extracted sensory-outcome table
Clark et al., 2019 [48]	RFFF	negative-pressure versus static-pressure dressings with split-thickness skin graft
Jaquet et al., 2012 [49]	RFFF	ulnar-based transposition flap versus split-thickness skin graft
De Lange et al., 2025 [50]	FFF	not reported or not sufficiently specified in extracted sensory-outcome table
Pototschnig et al., 2013 [51]	FFF	not reported or not sufficiently specified in extracted sensory-outcome table
Bartaire et al., 2012 [52]	FFF	not reported or not sufficiently specified in extracted sensory-outcome table
Attia et al., 2020 [53]	FFF	not reported or not sufficiently specified in extracted sensory-outcome table
Xu et al., 2017 [54]	FFF	not reported or not sufficiently specified in extracted sensory-outcome table
Maben et al., 2021 [55]	FFF	not reported or not sufficiently specified in extracted sensory-outcome table
Di Giuli et al., 2019 [56]	FFF	not reported or not sufficiently specified in extracted sensory-outcome table
Ali et al., 2021 [57]	FFF	not reported or not sufficiently specified in extracted sensory-outcome table
Celotti et al., 2020 [58]	FFF	not reported or not sufficiently specified in extracted sensory-outcome table
Lee et al., 2023 [59]	FFF	not reported or not sufficiently specified in extracted sensory-outcome table
Rashid et al., 2025 [60]	Both	not reported or not sufficiently specified in extracted sensory-outcome table
Cheon et al., 2025 [61]	Both	not reported or not sufficiently specified in extracted sensory-outcome table
Hidalgo, 1989 [62]	FFF (osseous and osteocutaneous)	not reported or not sufficiently specified in extracted sensory-outcome table
Anthony et al., 1995 [63]	FFF (osteocutaneous)	not reported or not sufficiently specified in extracted sensory-outcome table
Hidalgo et al., 1995 [64]	FFF (osseous 38%; osteocutaneous 62%; mandibular reconstruction only)	not reported or not sufficiently specified in extracted sensory-outcome table
Ferri et al., 1997 [65]	FFF	not reported or not sufficiently specified in extracted sensory-outcome table
Shpitzer et al., 1997 [66]	FFF	not reported or not sufficiently specified in extracted sensory-outcome table
Shpitzer et al., 1999 [67]	FFF	not reported or not sufficiently specified in extracted sensory-outcome table
Babovic et al., 2000 [68]	FFF	not reported or not sufficiently specified in extracted sensory-outcome table

Shindo et al., 2000 [69]	FFF (osteocutaneous; head and neck including tumour resection, trauma, osteoradionecrosis)	FFF wound-closure method comparison; sensory outcomes secondary
Zimmermann et al., 2001 [70]	FFF (osseous 38.1%; osteocutaneous 40.5%; osteomuscular 7.1%; osteomyocutaneous 14.3%)	not reported or not sufficiently specified in extracted sensory-outcome table
Muñoz Guerra et al., 2001 [71]	FFF (osteomuscular, with skin paddle in 24/26; 'double barrel' in 6 cases)	not reported or not sufficiently specified in extracted sensory-outcome table
Hidalgo & Pusic 2002 [72]	FFF (19/20 fibula; 1/20 scapula)	not reported or not sufficiently specified in extracted sensory-outcome table
Bodde et al., 2003 [73]	FFF (all included skin paddle)	not reported or not sufficiently specified in extracted sensory-outcome table
Garrett et al., 2006 [74]	FFF (osteomyocutaneous)	not reported or not sufficiently specified in extracted sensory-outcome table
Farhadi et al., 2007 [75]	FFF	not reported or not sufficiently specified in extracted sensory-outcome table
Lee et al., 2008 [76]	FFF	not reported or not sufficiently specified in extracted sensory-outcome table
González-García et al., 2008 [77]	FFF (osteocutaneous 90.4%; osseous 9.6%)	not reported or not sufficiently specified in extracted sensory-outcome table
Papadopulos et al., 2008 [78]	FFF osteofasciocutaneous	not reported or not sufficiently specified in extracted sensory-outcome table
Chou et al., 2009 [79]	FFF (osteoseptocutaneous)	not reported or not sufficiently specified in extracted sensory-outcome table
Sieg et al., 2010 [80]	FFF (osseous 37%, osteocutaneous 48%, osseomyocutaneous 10%, osseomuscular 5%)	not reported or not sufficiently specified in extracted sensory-outcome table
López-Arcas et al., 2010 [81]	FFF (osteocutaneous / osteomyocutaneous)	not reported or not sufficiently specified in extracted sensory-outcome table
Wolff et al., 2011 [82]	FFF (osteocutaneous 94%; osseous 6%; oral reconstruction — mandible 88%, maxilla 12%)	reduced tissue-alteration FFF harvest technique
Ling et al., 2013 [83]	FFF (19 patients) vs DCIA (15 patients)	not reported or not sufficiently specified in extracted sensory-outcome table
Rendenbach et al., 2016 [84]	FFF (mandibular reconstruction)	not reported or not sufficiently specified in extracted sensory-outcome table
Feuvrier et al., 2016 [85]	FFF	not reported or not sufficiently specified in extracted sensory-outcome table
Akashi et al., 2016 [86]	FFF osteocutaneous	primary closure versus skin grafting for FFF donor site

Supplementary Table S4. Sensitivity Analysis Summary

Outcome	Primary extractable-study estimate	Sensitivity excluding cohorts with n<20 and overlapping publications	Interpretation
RFFF sensory abnormality	Pooled 34.4%; median 31.3%; I2=88.4%	Estimate shifted downward and heterogeneity remained high.	Small cohorts and methodologically intensive outliers contribute to variability; descriptive medians remain important.
RFFF general pain	Pooled 16.0%; median 12.0%; I2=56.6%	Estimate changed modestly.	General pain estimates are less heterogeneous than sensory deficits but remain definition-dependent.
RFFF cold intolerance	Pooled 20.1%; median 16.2%; I2=50.2%	Estimate shifted downward after removing small cohorts.	Small mixed-flap and early studies influence upper range.
FFF sensory abnormality	Pooled 24.0%; median 21.4%; I2=88.6%	Estimate remained heterogeneous.	Objective testing and subjective symptom reports generate different prevalence estimates.
FFF general pain	Pooled 18.8%; median 17.5%; I2=77.7%	Estimate shifted downward after excluding small high-prevalence cohorts.	High pain rates in small cohorts should not be overinterpreted.
FFF cold intolerance	Pooled 15.0%; median 6.7%; I2=82.0%	Estimate shifted downward and heterogeneity remained.	Cold intolerance is inconsistently defined and often a secondary outcome.

Supplementary Table S5. Comparison with Prior Systematic Reviews

Review	Primary focus	How the present review differs
Bruin et al. 2022 [8]	RFFF neuropathic pain and altered sensation.	Adds FFF; updates literature; integrates DN4-based primary studies; adds objective/subjective methods comparison, risk-of-bias assessment, and exploratory quantitative summary.
Yalamanchili et al. 2020 [9]	Broad RFFF donor-site morbidity.	Focuses specifically on sensory abnormalities, pain phenotypes, cold intolerance, and assessment heterogeneity rather than overall morbidity alone.
Chen et al. 2016 [10]	ALT versus RFFF morbidity in tongue reconstruction.	Not limited to tongue reconstruction or ALT comparison; extracts donor-site sensory/pain outcomes across RFFF and FFF.
Loeffelbein et al. 2012 [11]	Evidence level for reducing RFFF donor-site morbidity.	Updates post-2012 evidence and appraises sensory/pain outcome definitions, not only donor-site morbidity reduction techniques.
Kearns et al. 2018 [12]	Osteocutaneous flap donor-site morbidity and PROMs.	Adds RFFF and FFF sensory/pain focus with methodological comparison of subjective versus objective sensory assessment.
Ling and Peng 2012 [13]	FFF donor-site morbidity broadly.	Updates FFF literature, incorporates neuropathic-pain screening data, and directly compares FFF with RFFF sensory outcome methodology.
